# An AI-powered patient triage platform for future viral outbreaks using COVID-19 as a disease model

**DOI:** 10.1186/s40246-023-00521-4

**Published:** 2023-08-29

**Authors:** Georgia Charkoftaki, Reza Aalizadeh, Alvaro Santos-Neto, Wan Ying Tan, Emily A. Davidson, Varvara Nikolopoulou, Yewei Wang, Brian Thompson, Tristan Furnary, Ying Chen, Elsio A. Wunder, Andreas Coppi, Wade Schulz, Akiko Iwasaki, Richard W. Pierce, Charles S. Dela Cruz, Gary V. Desir, Naftali Kaminski, Shelli Farhadian, Kirill Veselkov, Rupak Datta, Melissa Campbell, Nikolaos S. Thomaidis, Albert I. Ko, Yale IMPACT Study Team, Nathan Grubaugh, Allison Nelson, Anne L. Wyllie, Arnau Casanovas-Massana, Elizabeth B. White, Michael Chiorazzi, Michael Rainone, Rebecca Earnest, Sarah Lapidus, Joseph Lim, Maura Nakahata, Angela Nunez, Denise Shepard, Irene Matos, Yvette Strong, Kelly Anastasio, Kristina Brower, Maxine Kuang, M. Catherine Muenker, Adam J. Moore, Harold Rahming, Laura Glick, Erin Silva, Santos Bermejo, Pavithra Vijayakumar, Bertie Geng, John Fournier, Maksym Minasyan, Sean Bickerton, Melissa Linehan, Patrick Wong, Benjamin Goldman-Israelow, Anjelica Martin, Tyler Rice, William Khoury-Hanold, Jessica Nouws, David McDonald, Kadi-Ann Rose, Yiyun Cao, Lokesh Sharma, Mikhail Smolgovsky, Abeer Obaid, Giuseppe DeIuliis, Hong-Jai Park, Nicole Sonnert, Sofia Velazquez, Xiaohua Peng, Michael H. Askenase, Codruta Todeasa, Molly L. Bucklin, Maria Batsu, Alexander Robertson, Natasha Balkcom, Yicong Liu, Zitong Lin, Coriann Dorgay, Ryan Borg, Erendira Carmen Di Giuseppe, H. Patrick Young, Roy S. Herbst, David C. Thompson, Vasilis Vasiliou

**Affiliations:** 1 Department of Environmental Health Sciences, Yale School of Public Health, Yale University, New Haven, CT USA; 2 Laboratory of Analytical Chemistry, Department of Chemistry, National and Kapodistrian University of Athens, Athens, Zografou, 15771 Greece; 3 São Carlos Institute of Chemistry, University of São Paulo, São Carlos, SP 13566-590 Brazil; 4 Present Address: Internal Medicine Residency Program, Department of Internal Medicine, Norwalk Hospital, Norwalk, CT USA; 5 Department of Cellular and Molecular Physiology, Yale University School of Medicine, New Haven, CT USA; 6 Present Address: Harvard Medical School, Harvard University, Boston, MA USA; 7 Department of Epidemiology of Microbial Diseases, Yale School of Public Health, Yale University, New Haven, CT USA; 8 Institute Gonçalo Moniz, Fundação Oswaldo Cruz, Brazilian Ministry of Health, Salvador, Brazil; 9 Center for Outcomes Research and Evaluation, Yale-New Haven Hospital, New Haven, CT USA; 10 Department of Laboratory Medicine, Yale University School of Medicine, New Haven, CT USA; 11 Department of Immunobiology, Yale University School of Medicine, New Haven, CT USA; 12 Howard Hughes Medical Institute, MD Chevy Chase, USA; 13 Department of Pediatrics , Yale School of Medicine, New Haven, CT USA; 14 Section of Pulmonary, Critical Care and Sleep Medicine, Yale University School of Medicine, New Haven, CT USA; 15 Department of Internal Medicine, Section of Nephrology, Yale University School of Medicine, New Haven, CT USA; 16 Department of Internal Medicine, Section of Infectious Diseases, Yale School of Medicine, New Haven, CT USA; 17 Department of Neurology, Yale School of Medicine, New Haven, CT USA; 18 Department of Epidemiology of Microbial Diseases, Yale School of Public Health, Yale University, New Haven, USA; 19 Department of Surgery and Cancer, Imperial College London, South Kensington Campus, London, UK; 20 Veterans Affairs Connecticut Healthcare System, CT West Haven, USA; 21 Department of Internal Medicine, Yale School of Medicine, CT New Haven, USA; 22 Department of Pediatrics, Division of Pediatric Infectious Diseases, School of Medicine, Duke University, NC Durham, USA

## Abstract

Over the last century, outbreaks and pandemics have occurred with disturbing regularity, necessitating advance preparation and large-scale, coordinated response. Here, we developed a machine learning predictive model of disease severity and length of hospitalization for COVID-19, which can be utilized as a platform for future unknown viral outbreaks. We combined untargeted metabolomics on plasma data obtained from COVID-19 patients (n = 111) during hospitalization and healthy controls (n = 342), clinical and comorbidity data (n = 508) to build this patient triage platform, which consists of three parts: (i) the clinical decision tree, which amongst other biomarkers showed that patients with increased eosinophils have worse disease prognosis and can serve as a new potential biomarker with high accuracy (AUC = 0.974), (ii) the estimation of patient hospitalization length with ± 5 days error (R^2^ = 0.9765) and (iii) the prediction of the disease severity and the need of patient transfer to the intensive care unit. We report a significant decrease in serotonin levels in patients who needed positive airway pressure oxygen and/or were intubated. Furthermore, 5-hydroxy tryptophan, allantoin, and glucuronic acid metabolites were increased in COVID-19 patients and collectively they can serve as biomarkers to predict disease progression. The ability to quickly identify which patients will develop life-threatening illness would allow the efficient allocation of medical resources and implementation of the most effective medical interventions. We would advocate that the same approach could be utilized in future viral outbreaks to help hospitals triage patients more effectively and improve patient outcomes while optimizing healthcare resources.

**Supplementary Information:**

The online version contains supplementary material available at 10.1186/s40246-023-00521-4.

## Introduction

Most human coronavirus (CoV) infections result in mild patient symptoms. However, the novel severe acute respiratory syndrome coronavirus 2 (SARS-CoV-2) distinguishes itself from other CoVs in having led to more than 6.7 million deaths according to WHO (up to 23, January 2023. The outbreak of SARS-CoV-2 remains an on-going global pandemic. Elderly patients with underlying chronic diseases are considered at high risk for death from COVID-19, the disease caused by SARS-CoV-2, and younger people without major underlying diseases may also present with lethal complications [1].

Although vaccines appear to be safe and effective in preventing severe COVID-19 symptoms and death, the clinical management of COVID-19 patients continues to pose an enormous economic burden to the health system [2]. Patients with COVID-19 present a broad spectrum of symptoms ranging from asymptomatic to mild respiratory tract infections and influenza-like illness to severe disease with accompanying lung injury, multiorgan failure, and death [3]. Hypoxemia is a main marker of severity [4]. Although the lungs are believed to be the site at which SARS-CoV-2 replicates, infected patients often report other symptoms, suggesting the involvement of the gastrointestinal tract, heart, cardiovascular system, kidneys, and other organs [5].

Several studies have attempted to classify COVID-19 patients’ symptoms based on their clinical phenotypes [4, 6–8]. However, the heterogeneity in patient medical histories and COVID-19 symptoms have prevented the establishment of concrete classifications that can predict patient outcomes, i.e., who might need hospital admission or closer monitoring while in hospital. Being able to predict which patients can be sent home and those possibly needing intensive care unit (ICU) admission is critical to hospital administrators and health officials as they seek to implement interventions that optimize health outcomes for each COVID-19 patient and effectively utilize available hospital resources.

Using machine learning (ML), we built a model of COVID-19 disease severity and prediction of hospitalization duration based on clinical data and the metabolic profiles of plasma samples collected from patients during hospitalization. The model led us to identify a panel of unique clinical and metabolite biomarkers that were highly indicative of disease progression and allows the prediction of patient management needs very soon after hospital admission.

## Results

### Demographic data

The clinical cohort used in this study consisted of 431 participants, of whom 111 were considered SARS-CoV-2-positive upon admission in the Yale New Haven Hospital (YNHH). The remaining 324 participants were health care workers (HCW) healthy controls who were SARS-CoV-2-negative (Table 1). In sample collection, 131 of the samples from SARS-CoV-2-positive patients were collected from the same patient during their stay in the hospital. The SARS-CoV2 infection status of each study participant was confirmed by nasopharyngeal swab sample polymerase chain reaction (PCR) test. SARS-CoV-2-infected patients were arbitrarily divided into different classes based on their treatment needs during hospitalization as follows: classes 1 (patients who did not require any external oxygen supply), 2 (patients who required low or high flow oxygen supply), and 3 (patients who required positive airway pressure (biphasic; BIPAP or continuous; CPAP) or were intubated).

**Table 1:** Demographics and baseline characteristics of SARS-CoV-2-negative (uninfected) and -positive (infected) subjects

Variables	SARS-CoV-2-negative subjects	SARS-CoV-2-positive subjects			
	HCW^a^ Healthy Control (N = 324)	Total (N = 111)	Class 1^b^ (N = 29)	Class 2^c^ (N = 62)	Class 3^d^ (N = 20)
Sex-no. (%)					
Male	21.9	51.4	34.5	53.2	70.0
Female	78.1	48.6	65.5	46.8	30.0
Race (%)					
White/Caucasian	75.0	52.3	69.0	51.6	30.0
Black/African American	3.7	33.3	24.1	32.3	50.0
Asian	7.4	1.8	0.0	3.2	0.0
Other	4.9	11.7	6.9	12.9	15.0
Unknown	9.0	0.9	0.0	0.0	5.0
Ethnicity (%)					
Non-hispanic	80.6	82.0	79.3	83.9	80.0
Hispanic or Latino	8.6	17.1	20.7	16.1	15.0
Unknown	10.8	0.9	0.0	0.9	5.0
Age-year					
Mean (SD)	37.7 (11.4)	60.2 (18.2)	53.1 (18.8)	63.9 (16.8)	59.1 (19.1)
BMI (kg/m^2^)					
Mean (SD)	26.2 (5.5)	30.9 (8.9)	27.8 (7.5)	30.3 (8.5)	36.9 (9.7)
Time from admission to first positive PCR (days) (negative means PCR test prior to admission)					
Mean (SD)	N/A	−2.0 (7.9)	−5.0 (13.0)	−0.2 (3.9)	−3.3 (7.4)
Time from admission to advanced oxygen (days)					
Mean (SD)	N/A	N/A	N/A	N/A	3.4 (7.3)
Length of stay (days)					
Mean (SD)	N/A	14.2 (15.3)	6.3 (6.9)	14.6 (16.1)	24.5 (15.8)
SPO_2__24_hours_mean					
Mean (SD)	N/A	96.3 (1.7)	97.5 (1.3)	95.9 (1.5)	95.8 (1.9)
Survival (%)	100.0	93.7	100.0	100.0	65.0

^a^Healthy healthcare workers (HCWs, control).

^b^SARS-CoV-2-infected patients who during their hospitalization did not require any external oxygen supply.

^c^SARS-CoV-2-infected patients who required low or high flow oxygen supply.

^d^SARS-CoV-2-infected patients who required positive airway pressure (biphasic; BIPAP or continuous; CPAP) or were intubated.

The SARS-CoV2-infected cohort included a similar proportion of male and female patients (51.4 vs. 48.6%) (Table 1), and 52.3% were White/Caucasian, 33.3% Black/African American, 1.8% Asian, patients of other race were 11.7% and unknown 0.9%. For the healthy control group, 78.1% were female, 75% were White/Caucasian, 3.7% Black/African American, 7.4% Asian, other race 4.9% and unknown 9%. The mean (± SD) body mass index (BMI) and ages of the SARS-CoV2-infected cohort were 30.9 (± 8.9) kg/m^2^ and 60.2 ± 18.2 years of age, while the control cohort was at 26.2 ± 5.5 kg/m^2^ and 30.9 ± 8.9 years of age, respectively (Table 1). All SARS-CoV2-infected patients were admitted to the hospital.

### Untargeted metabolomics analyses between all SARS-CoV-2-infected patients and healthy controls

**Fig. 1:**
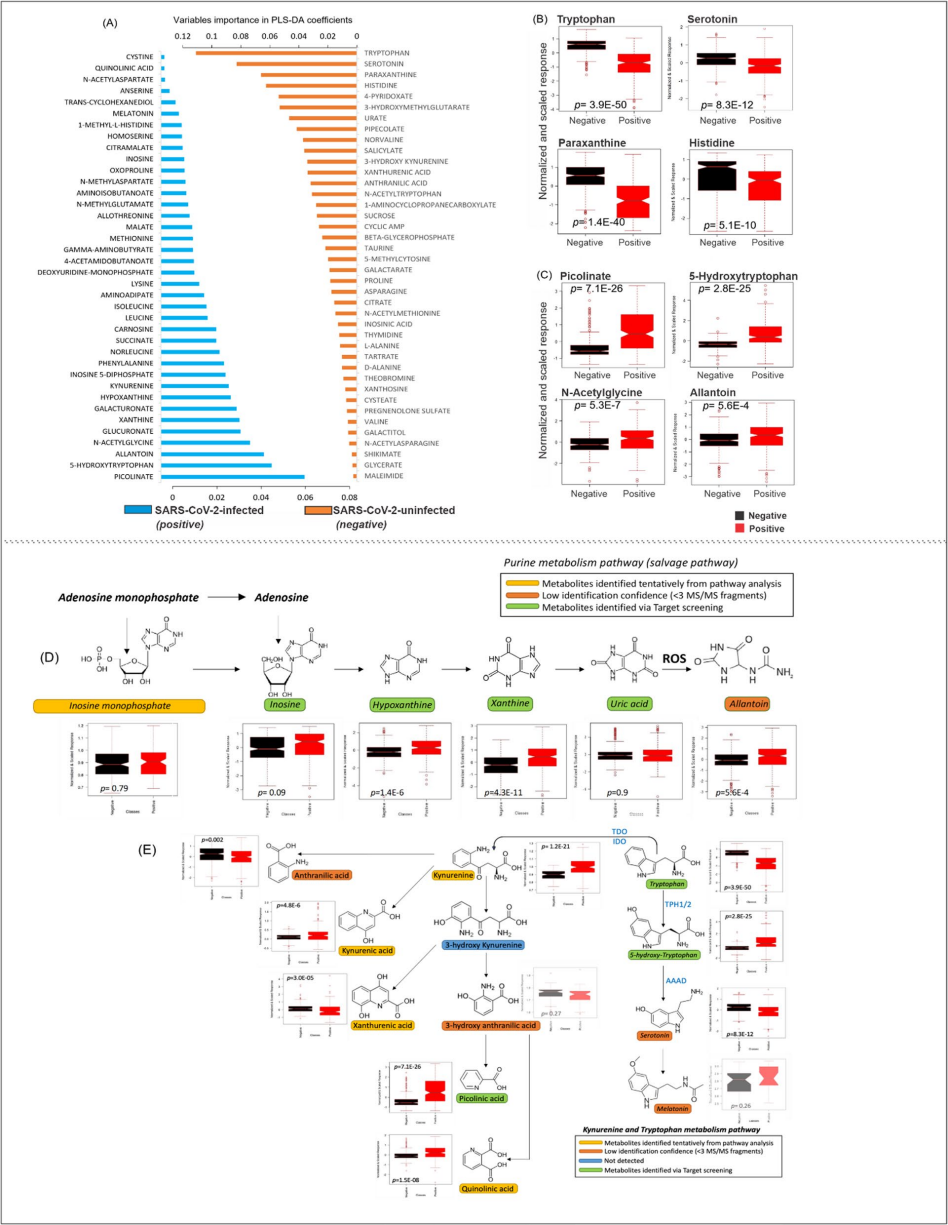
Plasma metabolome differences in SARS-CoV-2-infected and uninfected subjects. Order of importance of individual metabolites (in PLS-DA model) in SARS-CoV-2-uninfected (healthy control, orange bar) and SARS-CoV-2-infected (blue bar) subjects. **A** The four metabolites most significantly down-regulated in infected patients (relative to uninfected subjects). **B** The four most significant metabolites up-regulated in infected patients (red symbol) relative to uninfected subjects (black symbols). **C** Metabolic pathways identified by untargeted metabolomics in the plasma of SARS-CoV-2-uninfected subjects (black symbols) and SARS-CoV-2-infected patients (red symbols). **D** *Purine metabolism:* adenosine monophosphate can be converted to inosine either by (i) deamination to form inosine monophosphate followed by dephosphorylation or (ii) dephosphorylation to form adenosine followed by deamination. Hypoxanthine, formed from inosine, can undergo oxidative hydroxylation to xanthine which can then be converted by xanthine oxidase to uric acid. Allantoin is formed from the reaction between uric acid and reactive oxygen species (ROS). **E** *Tryptophan metabolism:* In the kynurenine pathway (which accounts for ~ 95% of tryptophan degradation), tryptophan forms kynurenine (by tryptophan-2,3-dioxygenase (TDO) or indoleamine 2,3-dioxygenase (IDO)). Kynurenine can then undergo hydroxylation to 3-hydroxy kynurenine (by kynurenine 3-monooxygenase (KMO)). A minor degradation pathway involves tryptophan hydroxylation to 5-hydroxy-tryptophan (by tryptophan hydroxylase isoforms 1 and 2 (TPH1/2)) and then to serotonin and melatonin (by aromatic-L-amino-acid decarboxylase (AAAD)). In figures **B–E**, data are presented as the mean ± SD and each dot represents individual sample results. Dots outside the box plot are in the upper quartile (75th percentile) of the distribution and the dots inside the box plot are in the interquartile range (IQR), where 50% of the data are located. Outside the box plot are the patients that are outside the IQR range. The box plot is divided at the median. Probability values reflect results in SARS-CoV-2-infected patients being compared with uninfected subjects using a Student’s unpaired t-test

Untargeted metabolomics was performed in plasma samples to elucidate whether SARS-CoV-2 infection caused changes in the plasma metabolic profiles of SARS-CoV-2 positive patients admitted to hospital. Comprehensive profiles were acquired and a total of 82 features were detected. Partial least square-discriminant analysis (PLS-DA) of the metabolomics data showed good separation of the metabolic profiles between the SARS-CoV-2-positive patients and the healthy controls (Additional file 1: Figure S1A). Multifactorial analyses revealed metabolite differences between all of the SARS-CoV-2-positive patients (class 1, 2, and 3) and the SARS-CoV-2-negative controls (HCW). Of these metabolites, picolinate was the metabolite that best predicted an individual infected by SARS-CoV-2, i.e., increased plasma levels occurred in infected patients (Fig. 1A, C, Additional file 1: Fig. S1B, D). By contrast, tryptophan was the plasma metabolite found to be associated with SARS-CoV-2-negative individuals (Fig. 1A, B, Additional file 1: Fig. S1B, E). Although many factors can cause variation in the plasma metabolome of the SARS-CoV-2-positive patients (e.g., underlying diseases, age, severity of COVID-19), the test samples showed similar parameters as the training set used in the PLS-DA model and were classified correctly as SARS-CoV-2-positive individuals (Additional file 1: Figure S1C). Multivariate and univariate analyses showed differences in the plasma metabolome between the two classes (uninfected vs. infected). The effect of the two most significant metabolites (picolinate and tryptophan) on the overall impact of SARS-CoV-2 infection and the estimation of probability values (which is discrimination criteria between SARS-CoV-2-positive and negative cases) is depicted in Additional file 1: Figures S1D, E. Receiver operating characteristic (ROC) curves were calculated for both the training and test sets and resulted in an area under curve value of 1.00. This demonstrates the robustness of the PLS-DA model and its applicability to the accurate discrimination between SARS-CoV-2-negative and SARS-CoV-2-infected individuals.

**Fig. 2:**
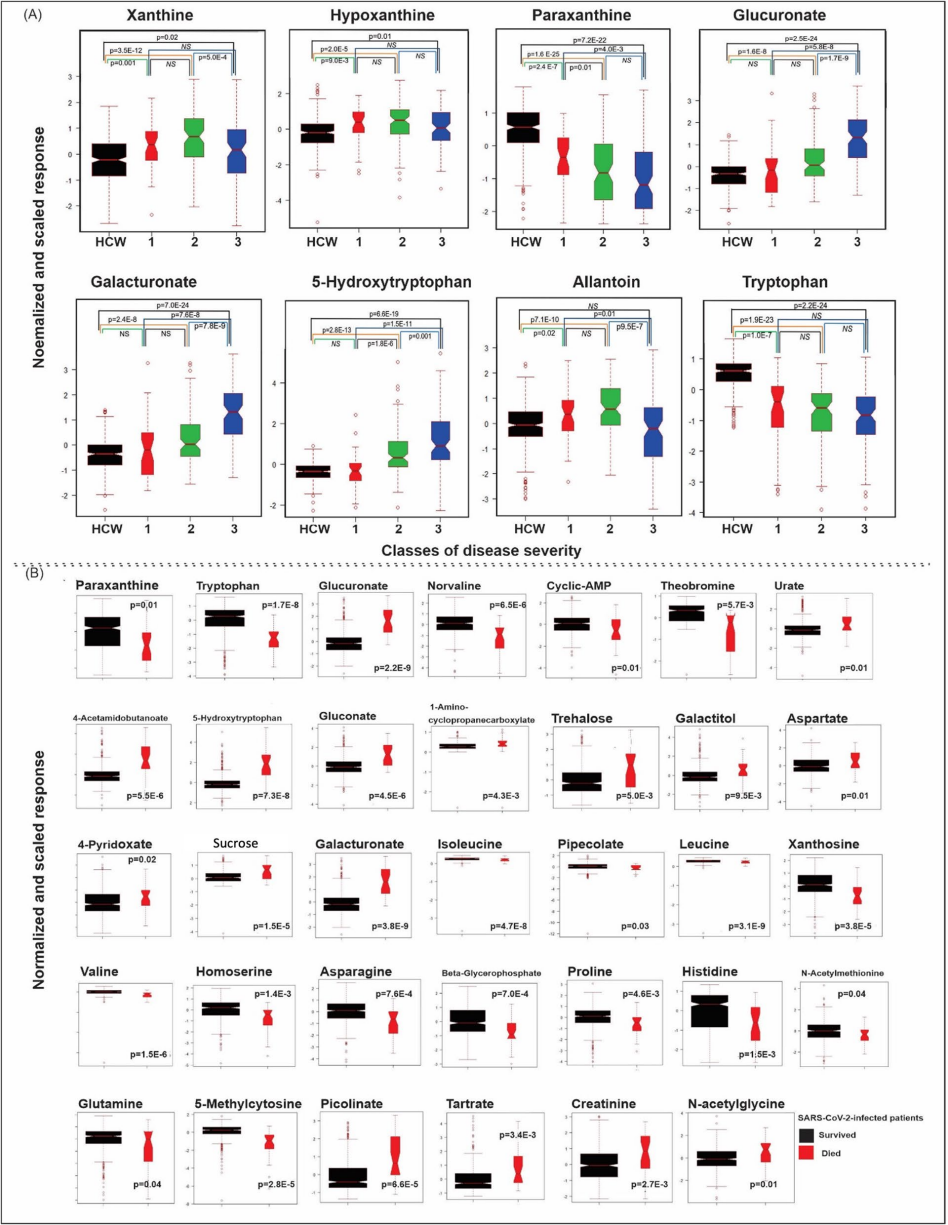
Untargeted plasma metabolomics analyses of SARS-CoV-2-infected and uninfected subjects. **A** Results of univariate analysis of the metabolites are shown for SARS-CoV-2-negative subjects (HCW black square), SARS-CoV-2-positive patients who during their hospitalization did not require any external oxygen supply or required only a low flow of oxygen (Class 1, red square), SARS-CoV-2-positive patients who required a high flow of oxygen (Class 2, green square), and SARS-CoV-2-positive patients who needed positive airway pressure (biphasic; BIPAP or continuous; CPAP) or were intubated (Class 3, blue square). **B** Univariate analysis of the identified metabolites in the plasma showing differences in the metabolome between SARS-Cov-2 infected patients who survived (black square) and those who did not survive (red square). Data are presented as the mean ± SD; dots outside the box plot are in the upper quartile (75th percentile) of the distribution and the dots inside the box plot are in the interquartile range (IQR), where 50% of the data are located. Outside the box plot are the patients that are outside the IQR range, and the box plot is divided at the median. Student’s unpaired t-test, NS (non-significant)  (color figure online)

Pathway analysis revealed metabolites changed in SARS-CoV-2-infected patients being involved in the purine salvage pathway. Plasma levels of inosine monophosphate, inosine or uric acid were not different between the healthy control and SARS-CoV-2-infected groups. However, hypoxanthine and xanthine were increased in SARS-CoV-2-infected patients (Figs. 1D, 2A) and univariate analysis also revealed significant changes for each class individually when compared to uninfected controls (Fig. 2). Similarly, allantoin, a metabolite derived from uric acid and reactive oxygen species [9], was higher in the SARS-CoV-2-infected patient group (Fig. 1A–D); however univariate analysis for the individual classes, revealed allantoin to be increased in classes 1 and 2 but not in class 3 patients when compared to uninfected controls (Fig. 2A).

The tryptophan metabolism pathway was also comprehensively characterized in the present study. In the SARS-CoV-2-infected patient group, plasma levels of tryptophan were decreased, while levels of 5-hydroxy-tryptophan and kynurenine were increased (Fig. 1B). By contrast, serotonin levels were decreased (Fig. 1B) in the SARS-CoV-2-infected group. Given that 5-hydroxy-tryptophan availability is the rate-limiting step in serotonin synthesis [10], the decreased serotonin levels observed in SARS-CoV-2-infected patients are unexpected. It is possible that SARS-CoV-2 infection may decrease plasma serotonin levels by reducing its synthesis from 5-hydroxy-tryptophan (e.g., by decreased expression or activity of aromatic L-amino acid decarboxylase (AAAD)), increasing its cellular uptake (e.g., by upregulation of the serotonin reuptake transporter) and/or increasing its metabolism (e.g., by increased activity or expression of metabolic enzymes, such as monoamine oxidase or carboxypeptidase A3). Which of these possibilities underlies the current observation remains to be established. Levels of kynurenine metabolites varied in SARS-CoV-2-infected patients in a manner that appeared to vary by pathway. Specifically, increased levels of kynurenic acid, picolinate and quinolonic acid occurred in the plasma of SARS-CoV-2-infected patients [11]. Notably, reduced levels of anthranillic acid and xanthurenic acid occurred in these same patients (Fig. 1E). Glucuronate plasma levels were increased in the SARS-CoV-2-infected patient group and levels of paraxanthine, a caffeine metabolism-related metabolite, were decreased in the SARS-CoV-2-infected patient group (Figs. 1D, 2). Univariate analysis of only the metabolome revealed thirty-four metabolites that were different in SARS-CoV-2 infected patients who survived compared to those who did not survive. Amongst them are metabolites that showed up in the pathway analysis (such as paraxanthine, tryptophan, and glucuronate) and new ones including norvaline, cyclic-AMP and theobromine (Fig. 2B).

### Machine learning: combined clinical, comorbidity and metabolomics data improved the prediction model

Initially, the analysis was performed using only patient clinical data during hospitalization (Additional file 1: Table S1) and comorbidities upon admission (Additional file 1: Table S2), an approach that resulted in lower total accuracy for the external data set (test set) (Additional file 1: Figure S2A, C). The combined clinical, comorbidity and metabolomics data improved the prediction accuracy of the model (Additional file 1: Figure S2B, D) from 0.916 to 0.954 and 0.938 to 0.953 (in case of intubation risk of classes 1 vs 2 and 2 vs 3, respectively). When modelling factors influencing the length of hospitalization, removal of metabolomic data decreased the squared correlation coefficient from 0.976 to 0.956. This supports the value of using combinatorial data instead of individual datasets (metabolomics versus clinical and comorbidity data). In our analyses, we used training data (which was a subset of 80% of the total dataset) to train the machine learning model, and testing data set (a subset of 20% of the total dataset) to independently evaluate the accuracy of the model. In these analyses, a DUPLEX algorithm [12] was used to select the cases in the test set because the selected cases had to be representative of training data for unbiased accuracy estimation.

### Clinical decision tree analysis: estimation of survival or mortality during hospitalization

**Fig. 3:**
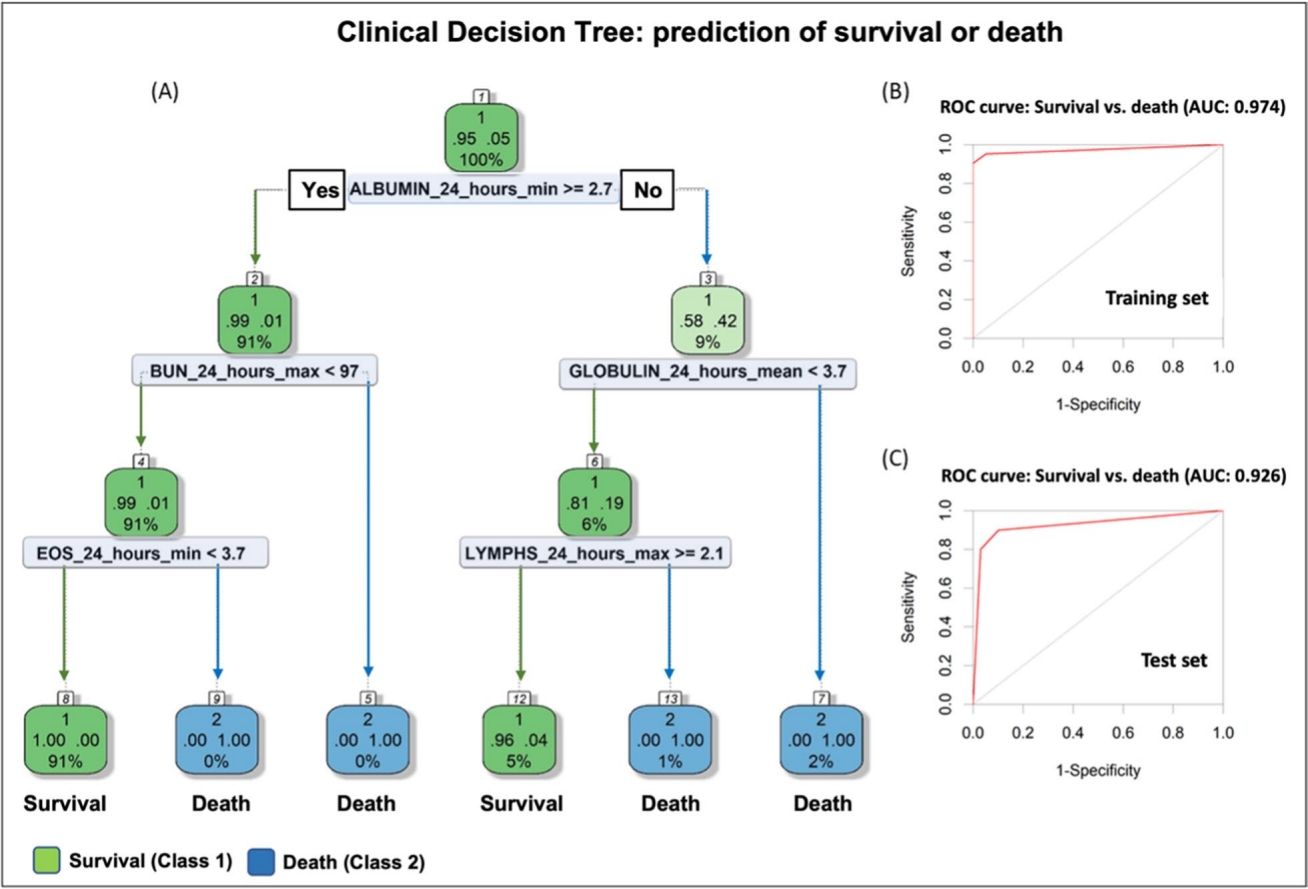
Clinical decision tree (DT). A clinical DT model predicting the discharge disposition of a patient (survival or death) was developed. **A** The tree shows the rules applied to classify each patient into the related classes (survival or death). At the top of the DT, the overall proportion of the patients survived (95%) or died (5%) is shown. Next, the node applies the threshold over clinical data to achieve classification of patients into the two classes. For instance, it applies the threshold of 2.7 g/dL over Albumin_24_hours_min (minimum value obtained from the clinical data), the node evaluates whether if patients show Albumin_24_hours_min above 2.7. If yes, then the next decision rule in DT is at down to the root’s left child node (Yes; depth 2). Ninety-one percent of patients will survive with a survival probability of ninety-nine percent. This way, inspecting the whole DT, the impact of features on the likelihood of survival can be derived. The percentage of patients at each node is provided below the probability values of survival (denoted as 1) or death (denoted as 2) on the DT; the green (survived) /blue (died) shows the fitted/estimated values for the patients in each class at given node. ROC curves for **B** training set and **C** test set. AUC provides an aggregate measure of performance across all possible classification thresholds

A clinical decision tree (DT) approach was developed from clinical and comorbidity data obtained during the SARS-CoV-2-infected patient’s hospital admission for the purpose of predicting the hospital death or survival (discharge disposition) of SARS-CoV-2-infected patients (Fig. 3A). The ROC curve for the training and test sets had accuracies of 0.974 and 0.926, respectively (Fig. 3B, C). The clinical DT has an internal feature selection method that includes factors that result in the lowest error towards estimation of discharge disposition. From both metabolomics and clinical data, clinical data were found to be sufficient to estimate the possibility of death or a survival event after SARS-CoV-2 infection at an early stage. Albumin was the initial determinant factor in the DT. If the albumin level is > 2.7 g/dL (normal range 3.5–5.5 g/dL) [13], then the blood urea nitrogen (BUN) should be considered. If BUN > 97 mg/dL (i.e., close to 5 times higher than the upper normal range of 20 mg/dL) [13], the clinical DT predicts that the patient will not survive (death). If the BUN < 97 mg/dL, the percentage of blood eosinophils (EOS) should be considered. If the EOS > 3.7, the clinical DT predicts that the patient would not survive; if the EOS < 3.7, the patient is predicted to survive. If the albumin level < 2.7 g/dL, then the globulin level should be evaluated. If globulin ≥ 3.7 g/dL, the patient is predicted not to survive; if globulin < 3.7 g/dL, the lymphocytes need to be determined to evaluate the discharge disposition of the patient. If lymphocytes $\ge$ 2.1%, the patient is predicted to survive; if lymphocytes < 2.1%, the patient is predicted not to survive (death).

### Prediction of duration of hospitalization

**Fig. 4:**
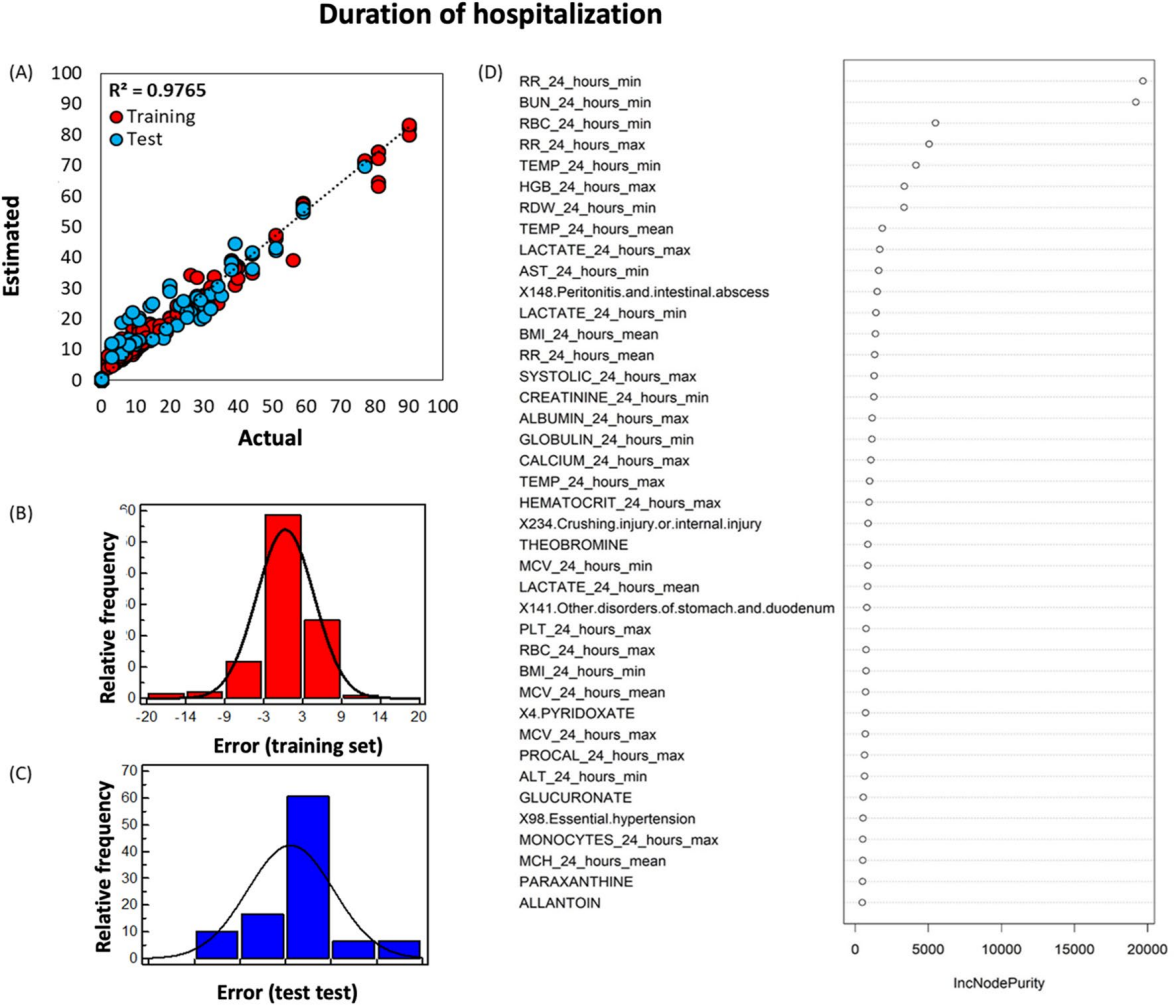
Random Forest (RF) machine learning algorithm to estimate the length of hospitalization of each SARS-CoV-2-infected patient admitted to the hospital. **A** Correlation plot between the RF estimated and the actual duration of hospitalization of SARS-CoV-2-infected patients; distribution of error (residuals) for training (**B,** red square) and test (**C,** blue square) sets. **D** The forty most significant factors in the structure of the RF model developed to predict the duration of hospitalization of SARS-CoV-2-infected patients (color figure online)

Through the application of machine learning analysis and the Random Forest (RF) regression to clinical, plasma metabolomics and comorbidity data, which we obtained during the hospitalization, the length of hospitalization of each SARS-CoV-2-infected patient admitted to the hospital was estimated. These estimates were found to be very accurate (i.e., with only few days of actual hospitalization duration), as shown in Fig. 4A (R^2^ = 0.9765). The error associated with more than 60% (≈ 1.5 sigma) of the data is within 3 to 5 days (Fig. 4B, C), indicating that the acceptable error window is ± 5 days. The forty most important factors in the structure of the RF model are depicted in Fig. 4D. Respiration (RR) was the most important factor contributing to the longer hospitalization of the patient. In the SARS-CoV-2-infected patient cohort, 73% had a RR above the normal of 18 breaths/min. Minimum blood urea nitrogen (BUN), a serum byproduct of protein metabolism, was the second critical clinical factor contributing to a prolonged hospitalization. Normal BUN ranges from 5 to 20 mg/dL [14] and in our cohort, 37% patients had BUN > 20 mg/dL. Considering the Shapley Additive exPlanations (SHAP) values between factors for patients with least and maximum duration of hospitalization (SARS-CoV-2-infected patient, patients #123 and #213 with 90- and 2-days duration of hospitalization, respectively), it can be concluded that simultaneous increases in BUN and RR values causes the duration of hospitalization to be increased (Additional file 1: Figure S3). Nevertheless, the cumulative effects of other factors apart from BUN and RR are essential to RF model to accurately estimate the duration of hospitalization. For example, for patient cases #123 and #213, excluding the other 508 factors can cause up to 20 and 8 days shifts in the outcome (Additional file 1: Figure S3). As expected, temperature was a factor to consider. Upon hospital admission, 36% SARS-CoV-2-infected patients had RBC < 4.0 million cells/μL (males and females; lower end of normal range for females [15]), 46% patients had HGB < 12 g/dL (males and females; lower end of normal range for females [15]). In 18.5% patients, PLT was lower than 150 (normal range: 150–400 × 10^9^/L [15]) and 44% had monocytes (differential, %) higher than 7% (normal range: 3–7%, [15],). Peritonitis and intestinal abscess were an underlying condition that was associated with prolonged hospitalization. Upon admission, only one patient had peritonitis/intestinal abscess, was hospitalized for nine days, intubated, and did not survive. During the present study, three more patients developed peritonitis/intestinal abscess. Of them, one patient stayed in the hospital for forty days, was intubated, and did not survive; the two other patients had a prolonged hospital stay for 77 and 90 days, both were intubated and survived. Fifty-nine (SARS-CoV-2-infected patients were male, and BMI data were obtained from fifty of them. Their mean BMI was 31.73 (8.62 SD) kg/m^2^ with six being in the healthy range (18–24.9 kg/m^2^). Of the fifty-six (SARS-CoV-2-infected female patients, BMI data were obtained from forty-seven. Their BMI was 30.77 (8.86 SD) kg/m^2^, with 25 being in the healthy range (25.0–29.9). Liver function tests (including aspartate aminotransferase (AST) and albumin) contributed to the estimation of the length of hospitalization. Sixty-three percent of the SARS-CoV-2-infected patients had AST values above the upper limit of normal (8-33U/L) [16], the albumin levels of 17% patients were lower than the lower limit of normal (3.5–5 g/dL) [17]. Lactate levels also contributed to estimation of the length of hospitalization, even though only 9 of 75 patients had hyperlactemia (lactate ≥ 2 mmol/L) [18] and we did not have data for 40 of the 111 patients. Systolic blood pressure data was available for 111 patients and 68 were found to have pressures exceeding 120 mmHg. The metabolites predicted by the model to be contributing to duration of hospitalization were (i) theobromine (a xanthine alkaloid and a product of caffeine metabolism) [19], (ii) glucuronic acid (a key metabolite of glucose involved in the detoxification of xenobiotic compounds which is produced in the liver) [20], (iii) paraxanthine (a metabolite of caffeine), and (iv) allantoin (a biomarker of oxidative stress in humans as the main product of uric acid oxidation by reactive oxygen species) [21] (Fig. 4D).

### Prediction of the risk of mechanical ventilation and/or intubation

**Fig. 5:**
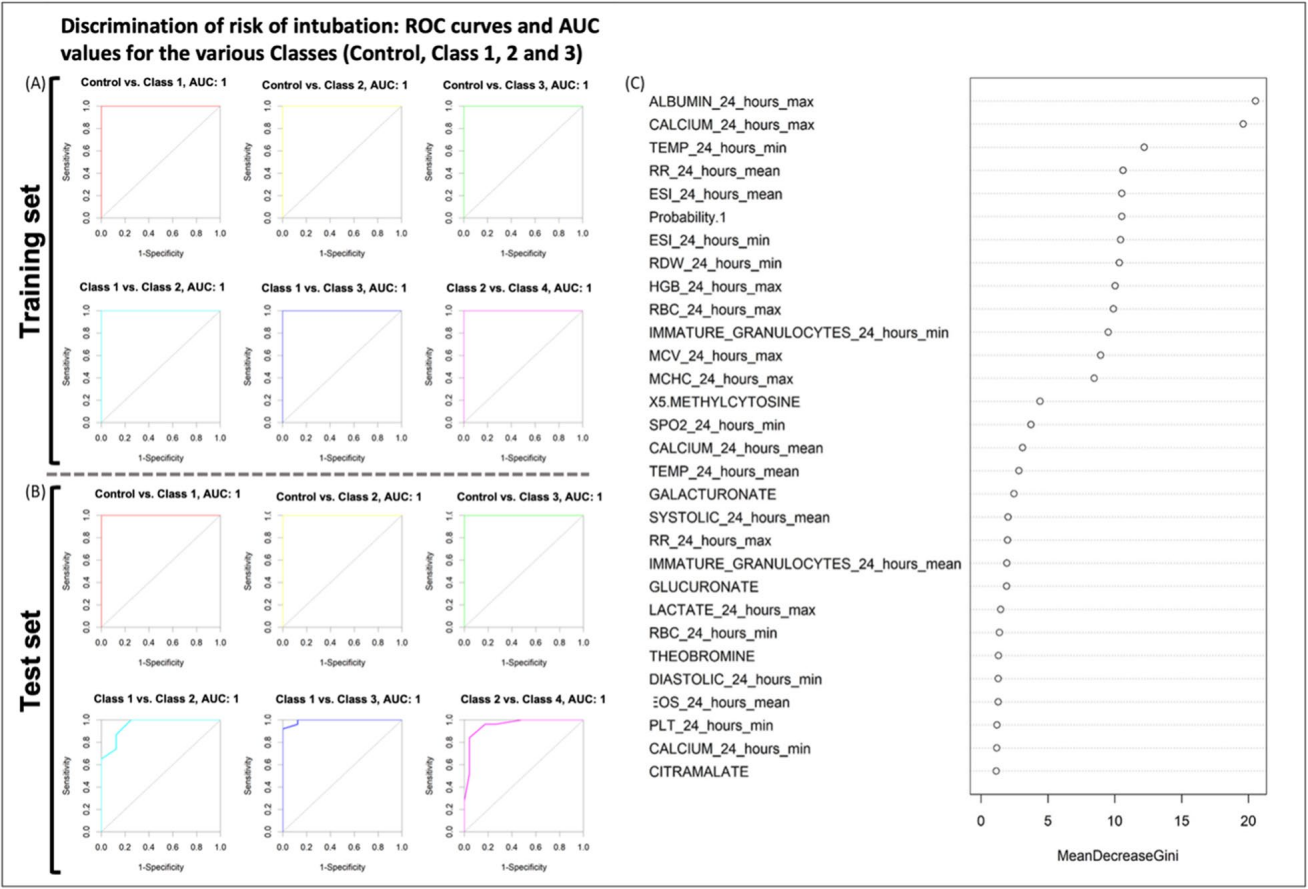
A pairwise comparison of classes by ROC curves in training (**A**) and test (**B**) sets; The ROC curves were derived pair-wise for the four risk classes, i.e., SARS-CoV-2-negative subjects (Control) SARS-CoV-2-positive patients who during their hospitalization did not require any external oxygen supply or required only a low flow of oxygen (Class 1), SARS-CoV-2-positive patients who required a high flow of oxygen (Class 2), and SARS-CoV-2-positive patients who needed positive airway pressure (biphasic; BIPAP or continuous; CPAP) or were intubated (Class 3). **C** The most significant factors in the structure of the Random Forest model developed to predict the risk of intubation due to SARS-CoV-2 infection. Note: Probability 1 (which is included in the most significant factors in the RF) is the output of the metabolomics results of the PLS-DA model showing the probability of healthy (Control) individuals

Machine learning analyses (similar to those described above) were performed to identify factors contributing to the risk of intubation of patients and to triage patients during hospitalization. Discrimination of risk of intubation was achieved for the three patient classes (with risk increasing from class 1 to class 3, with the AUC of the ROC curve being above 0.950 for pairwise comparison of the classes (Fig. 5A, B). This reflects the observation that fused data can be safely and reliably used to understand the risk of intubation in patients admitted to hospital with SARS-CoV-2 infection. Several factors found to be influential in the discrimination accuracy of the RF model included the following clinical data: albumin, calcium, temperature, respiration rate, ESI index (Emergency Severity Index: a triage tool for emergency departments; from clinical data), as well as plasma levels of 5-methylcytosine, galacturonate, glucuronate, theobromine and citramalate. (Fig. 5C).

### A personalized COVID-19 patient triage software application

Using the results obtained from our patient cohorts, a COVID Severity by Metabolomic and Clinical Study (CSMC) software was developed to support the pre-hospital process and to classify patients' condition when they arrive to the Emergency Department (Additional file 1: Figure S4). The software takes advantage of the power of machine learning and utilizes the clinical data (that are routinely obtained during hospital admission) and the plasma metabolomics data of the patient to predict: (i) the discharge disposition of the admitted patient (survival prediction), (ii) the length of hospitalization, and (iii) the disease prognosis (i.e., the risk of the patient for need for mechanical ventilation or intubation). The software supports a population health program for COVID-19 management by predicting care transitions, and patient monitoring during viral outbreaks. It can be accessed online here: http://trams.chem.uoa.gr/csmc/.

## Discussion

We performed pathway analysis and statistical analysis of the plasma metabolomics data and found them to be in agreement with previous publications [11, 22]; tryptophan metabolism is a pathway affected by SARS-CoV-2 infection. The tryptophan metabolic pathway was carefully characterized in the present study, and we found differences between the SARS-CoV-2 infected and uninfected groups, suggesting a disease-associated hyperactivation of the indoleamine-pyrrole 2,3-dioxygenase enzyme [23]. Activation of the kynurenine pathway was anticipated because it plays a major role in generation of cellular energy (in the form of nicotinamide adenine dinucleotide (NAD+)) which is increased substantially during an immune response. Higher levels of plasma kynurenine are associated with inflammation and psychiatric disorders [24]. This might explain the observed neurological disorders associated with the long-term effects of COVID-19. We also identified a decrease in plasma serotonin levels in SARS-CoV-2-infected patients who needed positive airway pressure oxygen and/or were intubated. Serotonin is an important autacoid and neurotransmitter. Studies have shown that SARS-CoV-2-infected patients treated with fluvoxamine, a selective serotonin reuptake inhibitor, had a lower likelihood of clinical deterioration [25, 26] and a very recent paper suggested that serotonin might be the missing link between COVID-19 course of severity in patients with diabetes and obesity [27]. Increased plasma levels in kynurenine, kynurenic acid, picolinic acid, and quinolinic acid (but not anthranilic acid) occurred in SARS-CoV-2-infected patients, suggesting hyperactivation of the kynurenine pathway in these subjects, as shown previously [28].

By combining the metabolomics results with the patient clinical data obtained during hospitalization, we developed an improved machine learning model compared to a model that only used clinical data. Our intent was to provide a personalized approach for SARS-CoV-2-infected patient management. Although some published machine learning models are used as screening, diagnostic [29] or prediction models of COVID-19 [30, 31], our model provides a real-time prediction of the clinical progression and duration of hospitalization based on both routinely-obtained clinical data and the plasma metabolomic profile.

Our clinical data findings are in agreement with those of Baker and coworkers [32]. Unlike previous assumptions [33], there appears to be a vigorous and early immune response in the upper airway in patients who develop COVID-19, a process that leads to recruitment of eosinophils, natural killer cells, and macrophages. In Baker's study, SARS-CoV-2-infected patients not treated with budesonide exhibited persistently raised interferon and eosinophil chemokines; patients with a worse disease prognosis showed a muted early inflammatory response (except for raised eosinophil chemokines), followed by a severe second peak of inflammation. In our clinical decision tree model, SARS-CoV-2-infected patients with increased eosinophils experienced worse clinical deterioration than those with lower eosinophil counts (< 3.7 cells/μL), leading to the possibility that blood eosinophil levels could serve as a potential biomarker for predicting the worsening of COVID-19.

In agreement with other studies [34, 35], we determined plasma albumin levels to be an important factor in predicting whether a SARS-CoV-2-infected patient would survive. In addition, it was the clinical parameter that exerted the most influence on the discrimination accuracy of the Random Forest model assessing the risk of intubation. It was also a factor predicting the duration of hospitalization. Albumin, the most abundant protein in plasma, performs important metabolic functions in the transport of free fatty acids, bilirubin, and many drugs [36]. Approximately 15 g of albumin is synthesized daily by the liver to maintain the albumin plasma steady state concentration [37]. Decreased albumin synthesis and increased catabolism after oxidation [38] is observed in liver disease and our dataset agrees with recent publications showing an association of hypoalbuminemia with poor outcome in critically ill population including COVID-19 [39].

The present study showed that inclusion of plasma metabolome data to the model improved the accuracy of the prediction of the duration of hospitalization of SARS-CoV-2-infected patients. Metabolites shown to be important in the model included allantoin, paraxanthine and theobromine, and glucuronic acid. Allantoin is an excellent biomarker of oxidative stress in humans [21, 40], and can be non-enzymatically oxidized from uric acid by reactive oxygen species (ROS). However, allantoin measurements are not routinely performed in clinical laboratories [21]. Many studies have demonstrated elevated levels of allantoin in a variety of diseases, including chronic heart failure [41], gout [42], and cystic fibrosis [43]. Methylxanthines, specifically paraxanthine and theobromine, which are found in coffee, cacao and tea [44], were found to be important in prediction of both the hospitalization duration and the severity of the disease. These metabolites are associated with consumption of cacao or caffeine-containing products, and thus can only be obtained through diet. As such, it is likely that the decreases in the levels of these metabolites in SARS-CoV-2-infected patients relates to decreased consumption of caffeine-containing or cacao-derived products. Interestingly, a recent study has suggested using methylxanthines to inhibit SARS-CoV-2 infection [45]. In the present study, glucuronic acid, a key metabolite of glucose involved in the detoxification of xenobiotic compounds [46], increased with the severity of SARS-CoV-2 infection symptoms and it contributed to both prediction of the hospitalization and the prognosis of the disease. A recent metabolomic study of patients with cirrhosis identified glucuronic acid as a biomarker of disease severity and future mortality [47], while another study demonstrated that glucuronic acid levels were robust predictors of all-cause mortality and correlate with future health span-related outcomes [20].

In the present study, large-scale plasma metabolomics analyses allowed us to identify metabolites, such as hydroxytryptophan, kynurenine, picolinic acid, allantoin and glucuronic acid, that were increased in SARS-CoV-2-infected patients. We also identified blood eosinophil count as a novel biomarker for COVID-19 disease severity. Combining all of the available clinical and comorbidity data with the metabolomics data, we demonstrated the value of metabolomics data to enhance model prediction. Further, the use of an advanced machine learning approach allowed the development a precision medicine model that is capable of being used for predicting outcomes in SARS-CoV-2-infected patients. This approach can be utilized for future viral outbreaks to help hospitals triage patients according to their need for emergency medical attention.

### Limitations

Our study has some limitations. First, it was conducted before vaccines were available and before many of the treatments that are available now, such as remdesivir, anti-SARS-CoV-2 monoclonal antibodies, and nirmatrelvir/ritonavir. One might expect such treatments would reduce the changes observed in our metabolite biomarkers. Second, the IMPACT study was conducted in a community setting in New Haven, CT (USA). This resulted in our population of healthy controls (i.e., health care workers) being mainly White/Caucasian, and despite our attempts to recruit ethnic minorities, the SARS-CoV-2-infected subjects comprised a higher proportion of Black/African Americans. As such, the possibility of race/ethnicity contributing to differences between SARS-CoV-2-infected and uninfected subjects cannot be excluded. These considerations notwithstanding, to our knowledge, this is the first study to combine both clinical and metabolomics data to build a model to predict hospitalization duration and disease severity.

## Materials and methods

### Chemicals

Ammonium acetate and ammonium hydroxide 25% v/v were purchased from Sigma-Aldrich (St. Louis, MO, USA), and acetonitrile and water (Optima® LC/MS grade) were purchased from Fisher Chemical (Fair Lawn, NJ, USA). Acetone, BAKER ANALYZED™ ACS Reagent, was obtained from J.T.Baker^®^ (VWR International, Pennsylvania, USA). For internal labeled standards, IROA^®^ TrueQuant IQQ Kit (IROA Technologies™, Sea Girt, NJ, USA) were used.

### Study design and participants

The cohort under study included plasma samples collected from SARS-CoV-2-infected patients during their hospitalization at the Yale New Haven Hospital (YNHH, n = 111) and from healthy (SARS-CoV-2-uninfected) Yale New Haven hospital healthcare workers (HCW, control) (n = 324) as part of the Yale IMPACT Biorepository. All samples were collected between March and May 2020 and all subjects in the study had not received any COVID-19 vaccinations. The healthcare workers were medical staff working in the YNHH. This time period preceded the availability of immunizations and medications for COVID-19. We included all patients with laboratory-confirmed SARS-CoV-2 infection who were hospitalized at the YNHH between March and May 2020. The protocol of this study was approved by the Institutional Review Board of Yale University (HIC number 2000027690). Written informed consent was obtained from all study participants. Subject demographic data are provided in Table 1. For data analysis, the subjects were divided into four groups as follows:

**Table:** 

Class	n	Characteristic
HCW	324	Healthy healthcare workers (control)
1	29	SARS-CoV-2-infected patients who during their hospitalization did not require any external oxygen supply
2	62	SARS-CoV-2-infected patients who required low or high flow oxygen supply
3	20	SARS-CoV-2-infected patients who required positive airway pressure (biphasic; BIPAP or continuous; CPAP) or were intubated

### Clinical and comorbidity data

The clinical data (Additional file 1: Table S1) were obtained during the patient hospitalization. They were retrieved from electronic medical records, including clinical characteristics (comorbidities, Additional file 1: Table S2), and laboratory test results assessing lung, kidney, liver, heart function, blood clotting/inflammation biomarkers, immune system, respiratory function, and metabolic panel. These were measured multiple times within the first 24 h of patient admission and we obtained a 24 h minimum value (24_hours_min), a 24 h maximum value (24_hours_max) and an average of all the measurements withing the 24 h window (24_hours_mean). In total, we obtained 65 different clinical parameters and had information for 281 different comorbidities according to the Emergency Severity Index. In addition, we had access to data regarding oxygen supply.

### Sample preparation

Untargeted metabolomic profiling was performed on plasma samples of SARS-CoV-2-infected and uninfected (healthy control) patients. The samples were prepared under BSL2 conditions using a protocol approved by the Yale Environmental Health and Safety Committee. The samples were selected for metabolite extraction in a randomized manner. Four hundred μL methanol:acetone (1:1% v/v) was added to 100 μL plasma, vortexed for 20 s and left at room temperature for 1 h for viral deactivation. The samples were then subjected to centrifugation (10,000 rpm) for 15 min at 4 °C. Four hundred μL of the resultant supernatant was aliquoted into an Eppendorf low binding tube (Protein LoBind® Tubes, Eppendorf US) and evaporated to dryness in a vacuum concentrator (ThermoFisher Scientific, Waltham, MA). Dry extracts were reconstituted in 120 μL acetonitrile:water (1:1, %v/v) containing 10 μL of IROA Internal Standard U-^13^C, 95% (IROA^®^ TrueQuant IQQ Kit, IROA Technologies™, Sea Girt, NJ, USA) and centrifuged (10,000 rpm) for 10 min at 4°C (to remove insoluble debris). One hundred μL of the supernatant was transferred into liquid chromatography-mass spectrometry vials (TrueView LC–MS Certified, Waters Corporation, Milford, MA) for LC–MS analysis.

Quality Control (QC) samples*:* Twenty μL of the sample supernatant was removed before the evaporation step. The aliquots were all pooled, and 400 μL aliquots were evaporated to dryness, stored and reconstituted as described above.

Untargeted metabolomic analysis of plasma samples: All extracted samples were analyzed on a quadrupole time-of flight (Q-ToF) mass spectrometer (Xevo G2-XS Q-ToF, Waters Corporation, Milford, MA) equipped with an ultra-performance liquid chromatography (UPLC) Acquity I Class (Waters Corporation, Milford, MA) unit. Chromatographic separation was performed using an Acquity BEH Amide column (particle size, 1.7 μm; 100 mm (length) × 2.1 mm (i.d.)) (Waters Corporation, Milford, MA) equipped with a BEH Amide VanGuard pre-column (5 × 2.1 mm, i.d.; 1.7μm) (Waters Corporation, Milford, MA) was used for chromatographic separation for Hydrophilic Interaction Liquid Chromatography (HILIC)-MS.

The mobile phase for HILIC-MS analysis consisted of A (25 mM ammonium hydroxide and 25 mM ammonium acetate in water) and B (acetonitrile) delivered at a flow rate of 0.5 mL/min. The linear gradient elution started at 95% B (0–0.5 min), 95%-65% B (0.5–7 min), 65–40% B (7–8 min), 40% B (8–9 min), 40–95% B (9–9.1 min) and continuing at 95% B (9.1–12.0 min). The injection volume for all samples and standard solutions was 3 μL. QC samples were analyzed every ten to fifteen injections. The column temperature was set at 30 °C for HILIC, and the sample tray temperature was maintained at 8 °C. For MS analyses, the electrospray ionization source (ESI) was operated in negative mode. Q-ToF–MS scan data (300 ms/scan; mass scan range 50–1200 Da) were first acquired for each sample. Thereafter, MS^e^ fragmentation data were acquired for metabolite identification (low energy scan: 200 ms/scan, collision energy 6 eV; high energy scan: 100 ms/scan, collision energy 10, 20, 30 and 40 eV, mass scan range 25–1000 Da). ESI source parameters were as follows: 1.8 kV capillary voltage, 40 V sampling cone, 50 °C source temperature, 420 °C desolvation temperature, 80 L/hr cone gas flow, 850 L/hr desolvation gas flow. Seven batches of randomized samples were run, and for each batch, a QC was injected 10 times before the individual samples for that sample type were injected to ensure matrix stabilization.

### Data analysis and structural annotation

ProteoWizard (version 3.06150) was used to convert raw MS data files to the .mzML format [48] and then imported in R environment for further analysis. A general target screening strategy outlined in our previous work [49] and the metabolite identification was performed using our in-house library. The target list was used in screening of MS^e^ data to confirm the metabolites. The screening strategy is depicted in Additional file 1: Figure S5. The screening was performed using in-house R algorithms based on several steps including:

1. Compiling list of our in-house library of metabolites with known MS^2^ fragments, experimental t_R_ (min) data, molecular formula and chemical identifier;
2. Creating extracted ion chromatogram (EIC) for the main adduct (50−) form calculated mass-to-charge ratio (m/z) value for the metabolite of interest;
3. Performing peak detection (if the peak is observable) and segmentation (to derive retention time range where the metabolite elutes) via a trained model based on convolutional neural network deep learning method (CNN-DL);
4. Checking contamination status for any peak presenting probability value > 0.5 (from step 3)) based on another CNN-DL based model [49]. Peaks showing a probability > 0.5 were considered to be a false positive and may exist in the sample due to analytical method or contamination (e.g., carry-over/presence in guard column, contamination in ion-source or during sample preparation);
5. Peaks with probability < 0.5 were checked for the existence of experimental isotopes (theoretically calculated from the molecular formula) and various adducts forms meaning that characteristic peaks for the given m/z at given mass accuracy (2 mDa) are available. This step uses the same algorithm used in step 3) to determine if characteristic peaks were present and provide a score of match;
6. Peaks showing enough evidence regarding the isotopes and adducts (i.e., score above 0.5) were evaluated by available retention time (t_R_) data. This step compared the t_R_ information between the t_R_ value in the database and the observed t_R_ for the sample. The threshold used on t_R_ value to accept the candidate was set to 20 s. If the absolute difference in the t_R_ value was above 30 s, the t_R_ data from the pooled sample (labeled TrueQuant metabolites spiked at a known concentration in the pooled samples) were used instead. Therefore, variation in t_R_ data due to matrix could be minimized, and if the absolute t_R_ difference became less than 30 s, the feature was kept; otherwise, it was moved to the list of unknowns (to be identified by non-target screening);
7. A candidate with acceptable absolute t_R_ difference was evaluated using available MS/MS fragments between initial database and samples. At this stage, the minimum number of three MS/MS fragments were required to be matched from the target database to the MS/MS data derived from sample using a doc product algorithm (threshold > 0.5) [50]. It is worth noting that structural isomers would not be distinguished using the current workflow unless their t_R_ data exceeded 30 s shifts.

Any detected compound (i.e., whose identification was confirmed through the above seven steps) was screened in all samples and the peak area values were extracted. The t_R_ range was obtained after using step 2) and the peak segmentation algorithm. Eventually, the metabolomics dataset included 559 samples (plus an additional 117 samples as QC), in which 82 metabolites were identified. The generated data used for machine learning consisted of the m/z value, t_R_, metabolite name and peak area.

### Machine learning analysis

To discriminate between plasma samples of SARS-CoV-2-uninfected and infected subjects, the dataset (consisting of metabolite name, m/z, t_R_, peak area and metabolite name for all samples (N = 559) was imported into the R environment and then transformed based on logarithmic (base 10) scale. In the metabolomics dataset, missing values were treated by predictive mean matching (PMM) algorithm using “MICE” R package [51]. The imputation of each dataset was performed groupwise (within class), i.e., the imputation was performed for metabolites by isolating them based on their group/class. Sixteen samples were excluded from the total list of 575 samples due to missing values. The complete data was then normalized by median value of each metabolite from the pooled samples (i.e., the QC set) as the estimation of the most probable quotient. The normalized dataset was then autoscaled prior to multivariate analysis. Subsequently, the dataset (a total of 559 samples without QC samples) was split into training (N = 449) and test set (N = 110) using the “DUPLEX” algorithm and “Mahalanobis” as the distance function [52, 53]. At first, Principal Component Analysis (PCA) was used to perform data exploratory analysis (using the “factoextra” and “mixOmics” R packages [54]). Partial least square discriminant analysis (PLS-DA) [54] was used as a supervised method to find markers that contribute to differentiation between samples from SARS-CoV-2-infected and uninfected patients. The optimal value for latent variables (LVs) used in PLS-DA was set by evaluating the misclassification error in 5-leave out cross-validation [55]. The statistical significance of the PLS-DA model was evaluated using R2X (which measures the accumulative variance), R2Y (which measures the goodness of fit), Q2 (fivefold-cross-validated; predictive ability of the model) and a Receiver Operating Characteristics (ROC) curve. Bayesian theorem was used to find threshold at which two classes were discriminated by PLS-DA [55]. This was needed to assign classes on the samples based on the calculated probability values. The threshold of absolute intensity was 0.0456 and was derived after evaluating the point at which specificity and sensitivity were equal to 1.00. Any probability value calculated using the PLS-DA method that lay above this 0.0456 threshold was class A (i.e., control; SARS-CoV-2-negative). Class B (i.e., SARS-CoV-2-positive) was assigned to a sample if the probability value was below 0.0456. The weight applied to each metabolite in each PLS-DA function was used as a basis to find potential markers that would discriminate between the samples. Outlier analysis and applicability domain were assessed using Hotelling’s T^2^ and Q residuals, respectively [55]. Any samples showing high Hotelling’s T^2^ and Q residual values were considered to be outliers. PLS-DA and related quality assessment factors were implemented in R and are available in “http://trams.chem.uoa.gr/csmc/”.

The risk of intubation of patients, the length of possible hospitalization, and patient discharge status from hospital were modelled using Random Forest (RF) [56]. In these analyses, the clinical, comorbidity data and scaled data of the metabolites were fused and used collectively. Overall, 510 factors (combined Additional file 1: Tables S1 and S2) were used in RF structure to model the disease severity. To enable rapid determination of COVID-19 severity, only clinical measurements (min, mean and max values of each test) measured for 24 h during their hospital admission were considered. This clinical Decision Tree (DT) represents a collection of individual DTs and it was trained on the basis of bootstrapped resampling (for regression case, such as length of hospitalization) and out-of-bag (OOB) misclassification error [56]. The risk of intubation was categorized into four groups on the basis of mechanical ventilation and/or intubation as follows: no risk (SARS-CoV-2-uninfected) (HCW), room air (class 1), low and high oxygen flow supplementation (class 2), and non-invasive (positive airway pressure, e.g., biphasic, BIPAP or continuous, CPAP) or invasive (intubation) oxygen supplementation (class 3). Discharge from hospital was a binary classification case: SARS-CoV-2-infected patients who survived and patients who died. The length of hospitalization was subjected to regression analysis and the uncertainty was calculated in terms of distribution of residuals. In addition, the quality of the models based on RF was evaluated internally and externally using training and independent test sets, and ROC curves. RF was implemented on the data using “rpart” and “randomForest” R packages. The interpretation of RF model was performed using SHAP and is based on game theory to explain the output of machine learning model. More details about SHAP can be found here [57]. SHAP was implemented in python using SHAP python package (https://pypi.org/project/shap/).

### Supplementary Information

**Additional file 1**. List of Supplementary Materials.

## Data Availability

All raw metabolomics data, along with the deconvoluted data will be deposited to the NIH Metabolomics workbench. The data will be linked to this publication for data search purposes. All other data are available in the main text or the supplementary materials.

## References

[1] Yang X (2020). Clinical course and outcomes of critically ill patients with SARS-CoV-2 pneumonia in Wuhan, China: a single-centered, retrospective, observational study. Lancet Respir Med.

[2] Richards F (2022). Economic burden of COVID-19: a systematic review. Clinicoecon Outcomes Res.

[3] Wang T, et al*.* Comorbidities and multi-organ injuries in the treatment of COVID-19. Lancet 395:e52. 10.1016/s0140-6736(20)30558-4 (2020). 10.1016/S0140-6736(20)30558-4 PMC7270177 32171074

[4] Rello J, Storti E, Belliato M, Serrano R. Clinical phenotypes of SARS-CoV-2: implications for clinicians and researchers. Eur Respir J. 2001028, 10.1183/13993003.01028-2020 (2020). 10.1183/13993003.01028-2020 PMC7236837 32341111

[5] Synowiec A, Szczepański A, Barreto-Duran E, Lie LK, Pyrc K. Severe acute respiratory syndrome coronavirus 2 (SARS-CoV-2): a systemic infection. Clin Microbiol Rev. 34. 10.1128/cmr.00133-20 (2021). 10.1128/CMR.00133-20 PMC7849242 33441314

[6] Lusczek ER. et al*.* Characterizing COVID-19 clinical phenotypes and associated comorbidities and complication profiles. PLoS One. 16:e0248956, 10.1371/journal.pone.0248956 (2021). 10.1371/journal.pone.0248956 PMC8011766 33788884

[7] Azoulay E (2020). Clinical phenotypes of critically ill COVID-19 patients. Intensive Care Med.

[8] Gutiérrez-Gutiérrez B (2021). Identification and validation of clinical phenotypes with prognostic implications in patients admitted to hospital with COVID-19: a multicentre cohort study. Lancet Infect Dis.

[9] Martinez-Moral M-P, Kannan K (2019). Allantoin as a marker of oxidative stress: inter- and intraindividual variability in urinary concentrations in healthy individuals. Environ Sci Technol Lett.

[10] Eteraf-Oskouei T, Najafi M. The relationship between the serotonergic system and COVID-19 disease: a review. Heliyon 8:e09544, 10.1016/j.heliyon.2022.e09544 (2022). 10.1016/j.heliyon.2022.e09544 PMC9132783 35652122

[11] Thomas, T., et al*.* COVID-19 infection alters kynurenine and fatty acid metabolism, correlating with IL-6 levels and renal status. JCI Insight. 5, 10.1172/jci.insight.140327 (2020). 10.1172/jci.insight.140327 PMC7453907 32559180

[12] Snee RD (1977). Validation of regression models: methods and examples. Technometrics.

[13] McPherson RA, Pincus MR. Henry's clinical diagnosis and management by laboratory methods, 24 edn (2021).

[14] Hosten AO. In Walker HK, Hall WD, Hurst JW, editors, Clinical methods: the history, physical, and laboratory examinations (Butterworths Copyright © 1990, Butterworth Publishers, a division of Reed Publishing., 1990). 21250045

[15] George-Gay B, Parker K. Understanding the complete blood count with differential. J Perianesth Nurs. 18:96–114; quiz 115–117, 10.1053/jpan.2003.50013 (2003). 10.1053/jpan.2003.50013 12710004

[16] https://www.mountsinai.org/health-library/tests/aspartate-aminotransferase-ast-blood-test

[17] Rozga J, Piątek T, Małkowski P (2013). Human albumin: old, new, and emerging applications. Ann Transpl.

[18] Bruno RR (2021). Lactate is associated with mortality in very old intensive care patients suffering from COVID-19: results from an international observational study of 2860 patients. Ann Intensive Care.

[19] Berends LM, van der Velpen V, Cassidy A (2015). Flavan-3-ols, theobromine, and the effects of cocoa and chocolate on cardiometabolic risk factors. Curr Opin Lipidol.

[20] Ho A (2019). Circulating glucuronic acid predicts healthspan and longevity in humans and mice. Aging (Albany NY).

[21] Kozlik P, Hasikova L, Stiburkova B, Zavada J, Kalikova K. Rapid and reliable HILIC-MS/MS method for monitoring allantoin as a biomarker of oxidative stress. Anal Biochem. 589:113509, 10.1016/j.ab.2019.113509 (2020). 10.1016/j.ab.2019.113509 31747555

[22] Sindelar, M., et al. Longitudinal metabolomics of human plasma reveals prognostic markers of COVID-19 disease severity. Cell Rep Med 2:100369, 10.1016/j.xcrm.2021.100369 (2021). 10.1016/j.xcrm.2021.100369 PMC8292035 34308390

[23] Acovic A (2018). Role of indoleamine 2,3-dioxygenase in pathology of the gastrointestinal tract. Therap Adv Gastroenterol.

[24] Halaris A (2017). Inflammation-associated co-morbidity between depression and cardiovascular disease. Curr Top Behav Neurosci.

[25] Lenze EJ (2020). Fluvoxamine vs placebo and clinical deterioration in outpatients with symptomatic COVID-19: a randomized clinical trial. JAMA.

[26] Seftel D, Boulware DR. Prospective cohort of fluvoxamine for early treatment of coronavirus disease 19. Open Forum Infect Dis 8:ofab050, 10.1093/ofid/ofab050 (2021). 10.1093/ofid/ofab050 PMC7888564 33623808

[27] Santos AP, Couto CF, Pereira SS, Monteiro MP (2022). Is serotonin the missing link between COVID-19 course of severity in patients with diabetes and obesity?. Neuroendocrinology.

[28] Cihan M, et al. Kynurenine pathway in Coronavirus disease (COVID-19): potential role in prognosis. J Clin Lab Anal. 36, e24257. 10.1002/jcla.24257 (2022). 10.1002/jcla.24257 PMC8906035 35092710

[29] Bendavid I (2022). A novel machine learning model to predict respiratory failure and invasive mechanical ventilation in critically ill patients suffering from COVID-19. Sci Rep.

[30] Mueller YM (2022). Stratification of hospitalized COVID-19 patients into clinical severity progression groups by immuno-phenotyping and machine learning. Nat Commun.

[31] Shiri I (2022). High-dimensional multinomial multiclass severity scoring of COVID-19 pneumonia using CT radiomics features and machine learning algorithms. Sci Rep.

[32] Baker JR (2022). Early Th2 inflammation in the upper respiratory mucosa as a predictor of severe COVID-19 and modulation by early treatment with inhaled corticosteroids: a mechanistic analysis. Lancet Respir Med.

[33] Mason RJ (2020). Thoughts on the alveolar phase of COVID-19. Am J Physiol Lung Cell Mol Physiol.

[34] Xu Y (2021). Serum albumin levels are a predictor of COVID-19 patient prognosis: evidence from a single cohort in Chongqing. China Int J Gen Med.

[35] Turcato G (2022). Severity of SARS-CoV-2 infection and albumin levels recorded at the first emergency department evaluation: a multicentre retrospective observational study. Emerg Med J.

[36] Sleep D (2015). Albumin and its application in drug delivery. Expert Opin Drug Deliv.

[37] Levitt DG, Levitt MD (2016). Human serum albumin homeostasis: a new look at the roles of synthesis, catabolism, renal and gastrointestinal excretion, and the clinical value of serum albumin measurements. Int J Gen Med.

[38] Lapenna D (2022). Regarding: hypoalbuminemia in COVID-19. J Intern Med.

[39] Zekri-Nechar, K., et al. Albumin binds COVID-19 spike 1 subunit and predicts in-hospital survival of infected patients-possible alteration by glucose. J Clin Med 11. 10.3390/jcm11030587 (2022). 10.3390/jcm11030587 PMC8836760 35160039

[40] Kand'ár R, Záková P (2008). Allantoin as a marker of oxidative stress in human erythrocytes. Clin Chem Lab Med.

[41] Caussé E, Fournier P, Roncalli J, Salvayre R, Galinier M (2017). Serum allantoin and aminothiols as biomarkers of chronic heart failure. Acta Cardiol.

[42] Kurajoh M (2012). Relationship between serum allantoin and urate in healthy subjects and effects of benzbromarone in gout patients. Int J Clin Pharmacol Ther.

[43] Dickerhof N (2017). Oxidized glutathione and uric acid as biomarkers of early cystic fibrosis lung disease. J Cyst Fibros.

[44] Camandola S, Plick N, Mattson MP (2019). Impact of coffee and cacao purine metabolites on neuroplasticity and neurodegenerative disease. Neurochem Res.

[45] Rolta R (2022). Methylxanthines as potential inhibitor of SARS-CoV-2: an in silico approach. Curr Pharmacol Rep.

[46] Fujiwara R, Yoda E, Tukey RH (2018). Species differences in drug glucuronidation: Humanized UDP-glucuronosyltransferase 1 mice and their application for predicting drug glucuronidation and drug-induced toxicity in humans. Drug Metab Pharmacokinet.

[47] Mindikoglu AL (2018). Unique metabolomic signature associated with hepatorenal dysfunction and mortality in cirrhosis. Transl Res.

[48] Chambers MC (2012). A cross-platform toolkit for mass spectrometry and proteomics. Nat Biotechnol.

[49] Nikolopoulou V, Aalizadeh R, Nika M-C, Thomaidis NS. TrendProbe: time profile analysis of emerging contaminants by LC-HRMS non-target screening and deep learning convolutional neural network. J Hazard Mater 428:128194, 10.1016/j.jhazmat.2021.128194 (2022). 10.1016/j.jhazmat.2021.128194 35033918

[50] Bletsou AA, Jeon J, Hollender J, Archontaki E, Thomaidis NS (2015). Targeted and non-targeted liquid chromatography-mass spectrometric workflows for identification of transformation products of emerging pollutants in the aquatic environment. TrAC Trends Anal Chem.

[51] van Buuren S, Groothuis-Oudshoorn K (2011). mice: multivariate imputation by chained equations in R. J Stat Softw.

[52] Daszykowski M, Walczak B, Massart DL (2002). Representative subset selection. Anal Chim Acta.

[53] Daszykowski M (2007). TOMCAT: a MATLAB toolbox for multivariate calibration techniques. Chemom Intell Lab Syst.

[54] Rohart F, Gautier B, Singh A, Lê Cao K-A. mixOmics: An R package for ‘omics feature selection and multiple data integration. PLOS Comput Biol 13:e1005752, 10.1371/journal.pcbi.1005752 (2017). 10.1371/journal.pcbi.1005752 PMC5687754 29099853

[55] Ballabio D, Consonni V. Classification tools in chemistry. Part 1: linear models. PLS-DA. Anal Methods 5:3790–8, 10.1039/C3AY40582F (2013).

[56] Breiman L (2001). Random forests. Mach Learn.

[57] Lundberg SM (2020). From local explanations to global understanding with explainable AI for trees. Nate Mach Intell.

