# Supplementary material for: An AI-powered patient triage platform for future viral outbreaks using COVID-19 as a disease model
Source: Hum Genomics. 2023 Aug 29;17:80. doi: 10.1186/s40246-023-00521-4 (PMC10463861; doi:10.1186/s40246-023-00521-4)
Supplement: Supplementary file 1 — Additional file 1. List of Supplementary Materials. [file 40246_2023_521_MOESM1_ESM.docx]

**List of Supplementary Materials**

Fig. S1 to S5

**
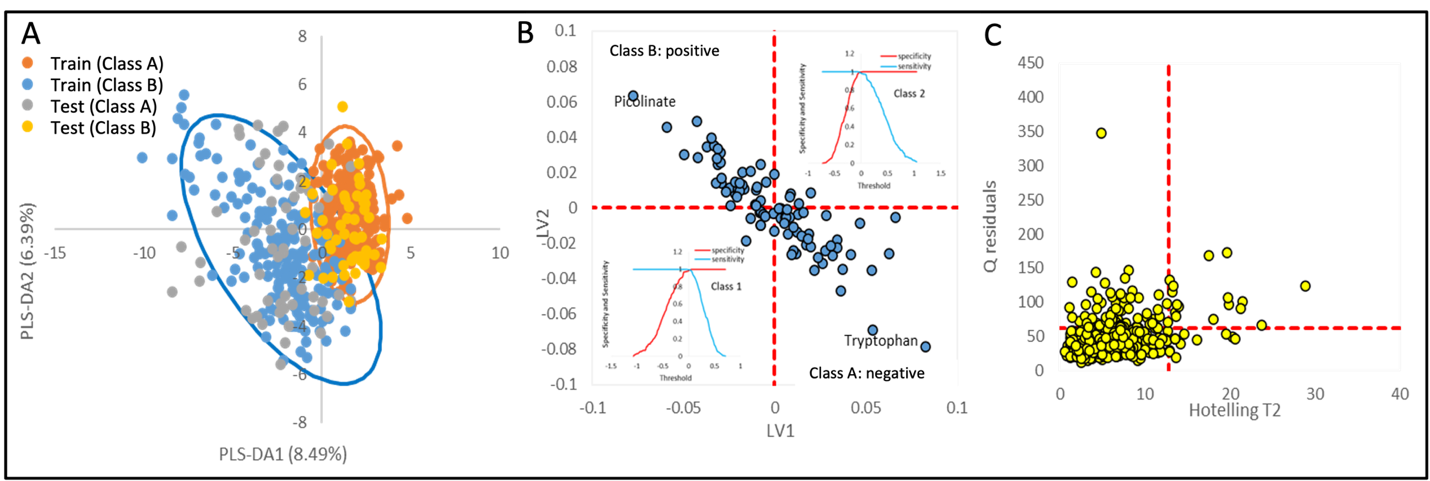
**


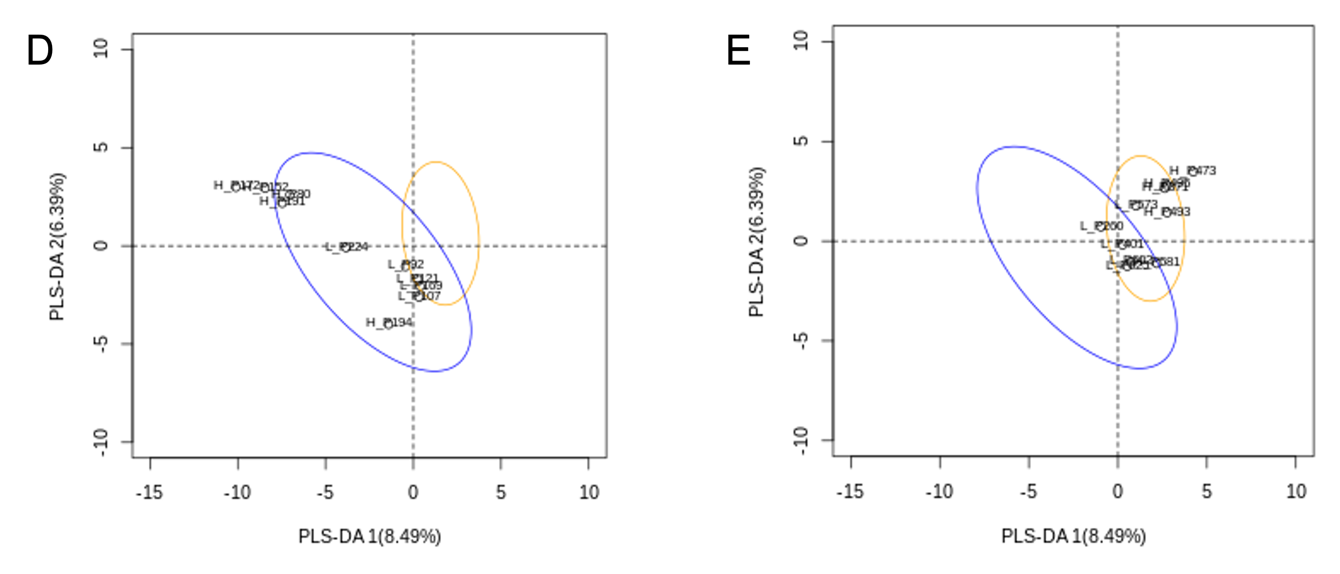


**Figure S1. Partial Least Square-Discriminant Analysis (PLS-DA) model to predict an individual infected by SARS-CoV-2.** (**A**) The discrimination between SARS-CoV-2-positive and healthy control cases with confidence interval around each class (95% confidence interval (CI)); In this plot, train and test refers to split of initial database into two subsets which used to develop the model (Train set) and then evaluate its accuracy with an evaluation set (test). In addition, Class A and B refers to HCW and SARS-COV-2-positive cases respectively, which were used as a binary classification problem in the PLS-DA model. The eclipse (confidence interval) around each class shows the area where the class assignment for a suspect case can be achieved with 95% CI by PLS-DA model (**B**) PLS-DA coefficients and threshold derived from Bayesian theorem to assign class information on suspected case (threshold 0.0456 at which the specificity and sensitivity equal to 1.00 which is translated into b1 and b2 (coefficients in the PLS-DA model) dimension for finding metabolites that are important towards each class); (**C**) outlier analysis based on Hotelling’s T2 versus Q residuals plot. The plots **D** and **E** show the impact of SARS-COV-2 on the metabolome and more specifically how the levels of picolinate (**D**) and tryptophan (**E**) can predict the patient’s classification: SARS-COV-2 positive or negative. The red and blue elliptical regions are the 95% CI confidence interval around HCW and SARS-COV-2-positive cases, respectively. The changes between low (denoted by “L” on the plot) and high (denoted by “H” on the plot) normalized peak area values for the metabolite is associated with significant changes in the probability values in PLS-DA outcome. As can be seen, the lower the normalized peak area becomes for picolinate, the cases scatter closer to confidence interval of SARS-COV-2-negative cases. (**E**) Similarly, the effect of tryptophan on the classification of SARS-COV-2 cases is depicted here: The lower the normalized peak area becomes for tryptophan, the cases scatter closer to the confidence interval of SARS-COV-2-positive cases and vice versa.


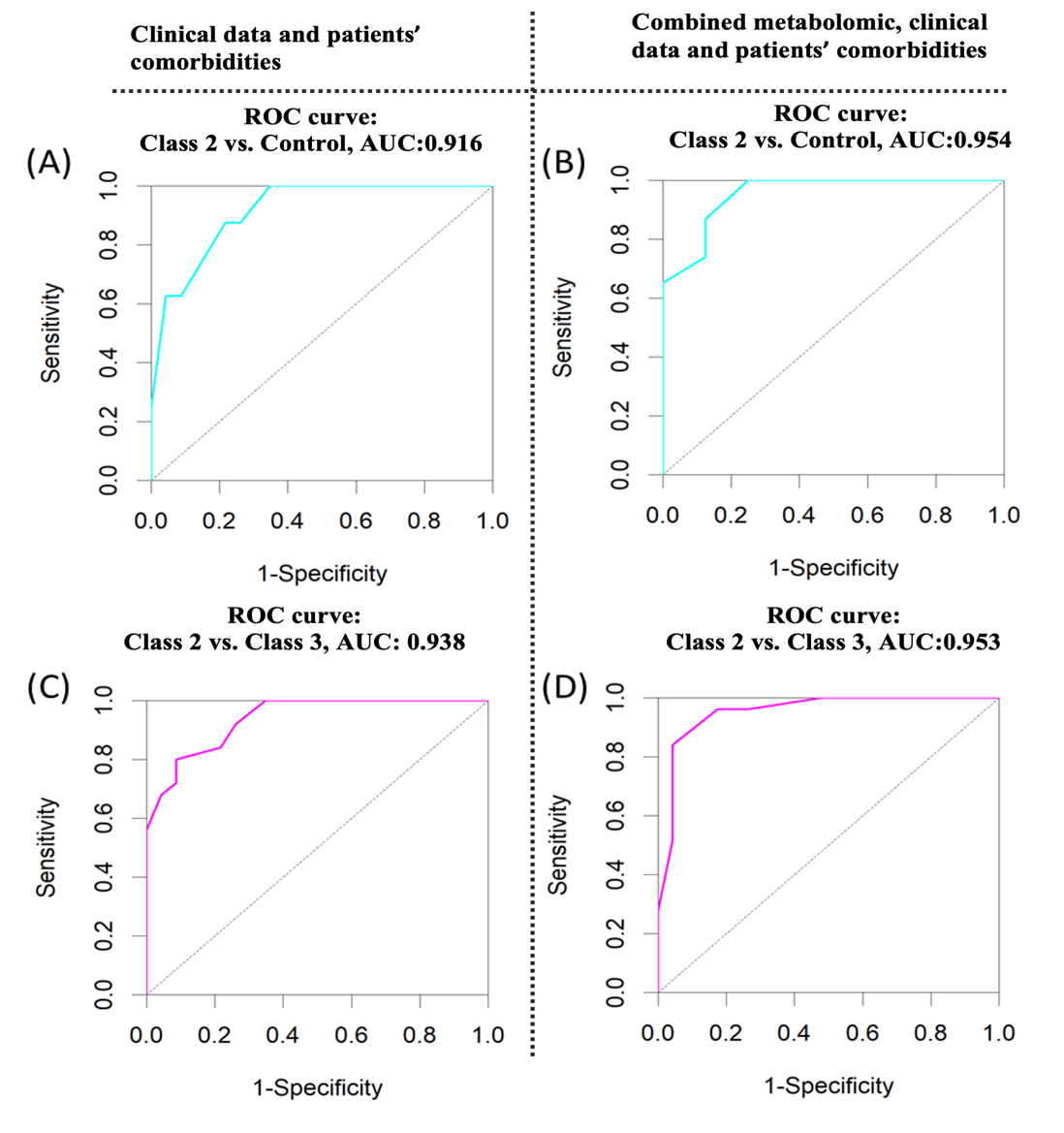


**Figure S2**. **ROC curves for relative intubation risk for SARS-CoV-2 infected patients** **(A)**: only clinical and comorbidity data were included in the modeling (combined Table S1, S2) to compare class 2 (low and high flow oxygen supply) vs. control, **(B)**: both metabolomic, clinical and comorbidity data were used during modeling and classes 2 vs. control, **(C)**: only clinical and comorbidity data were included in the modeling class 2 vs. class 3 (positive pressure airway oxygen supply and/or intubation), **(D)**: both metabolomic, clinical data and were used during modeling class 2 vs. class 3. The ROC curves show the drop in total accuracy when the metabolomics data were excluded from modeling process.


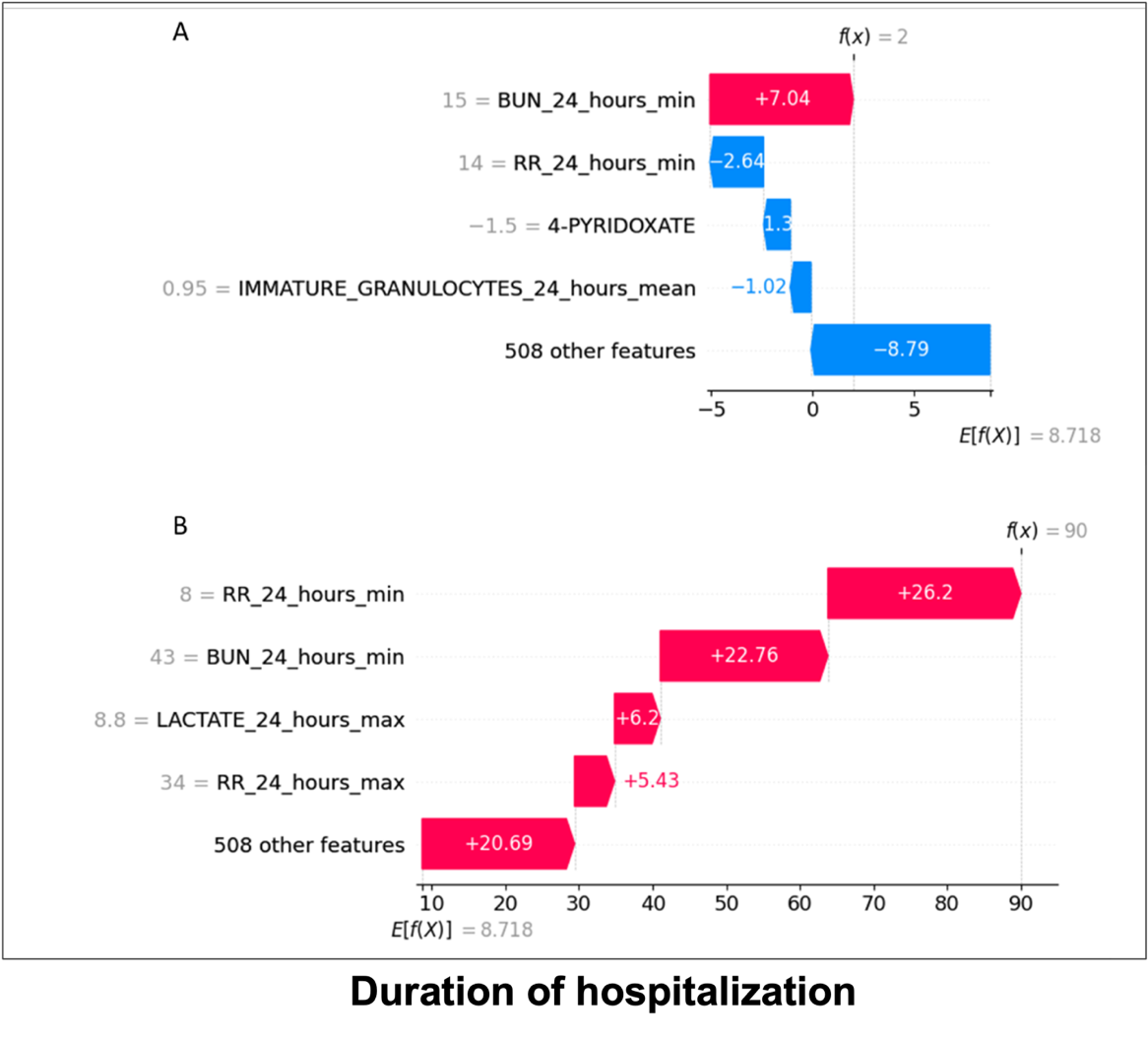


**Figure S3. Impact of metabolomic, clinical and comorbidity variables on Shapley Additive exPlanations (SHAP) values.** SHAP values are presented for the two SARS-CoV-2-infected patient cases with the lowest and highest duration of hospitalization, i.e., 2 days (patient #213, **A**) and 90 days (patient #123, **B**). In this plot, the effect of an individual variable is interpreted on the outcome of RF model. Red bars show the additive property of a related variable. Blue bars show that addition of a related variable decreases the base value estimated by model. This analysis allows the effect of the most important factor for an individual sample to be interpreted. For example, an increase in BUN causes the base value of 8.7 days (initially estimated by the model) to be increased by +22.8 days (B). f(X) shows the duration of hospitalization and E[f(x)] is the base estimation of duration of hospitalization. The value of each variable for each patient is provided to the left of the clinical variable associated with each red or blue bar. This plot also demonstrates that the accumulative effect of other variables is essential to accurately estimate the duration of hospitalization.


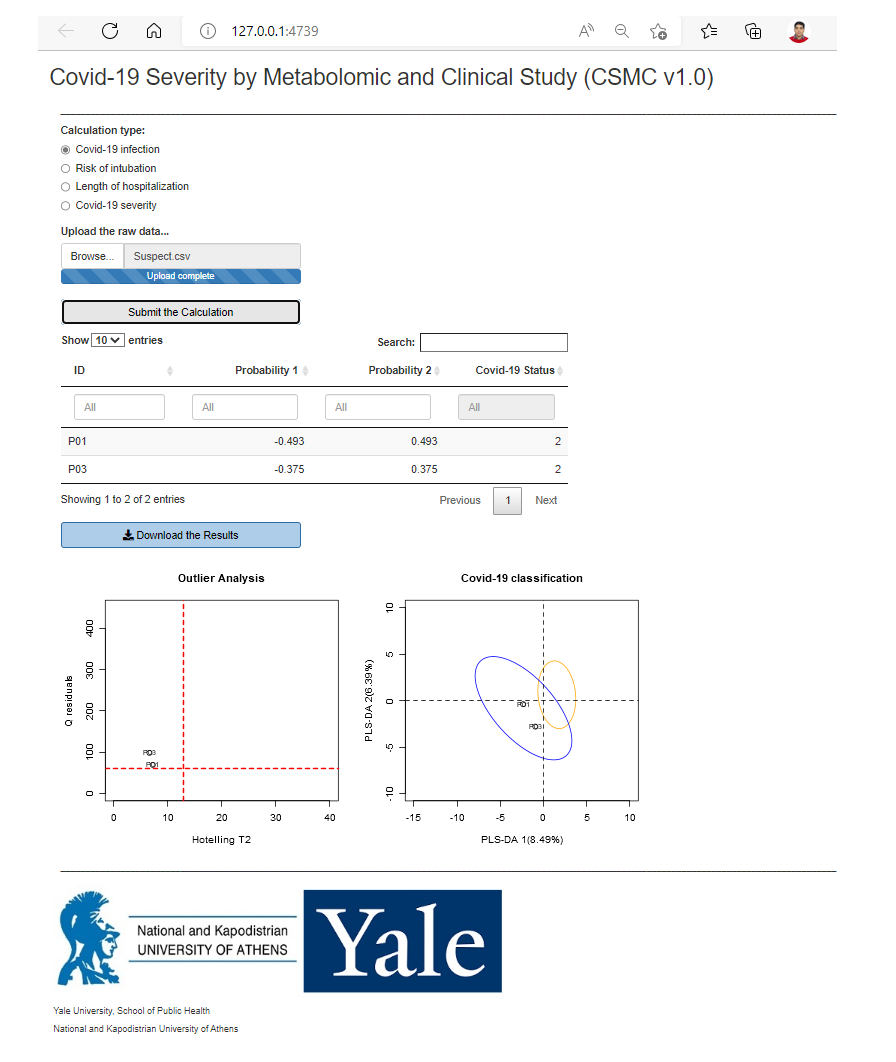


**Figure S4.** Representative image of the COVID Severity by Metabolomic and Clinical Study (CSMC) software which was developed to support the pre-hospital process and to classify patients' condition when they are admitted to the hospital and the software can be accessed online here: http://trams.chem.uoa.gr/csmc/.


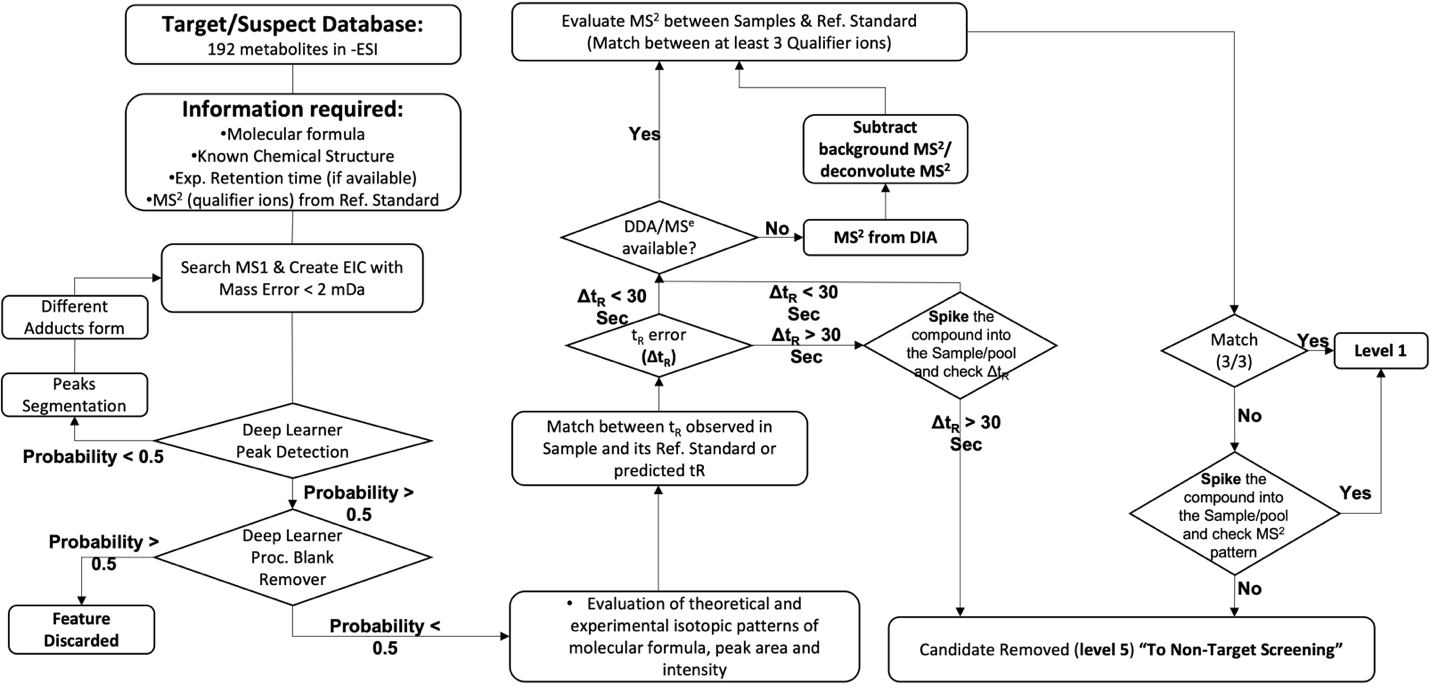


**Figure S5.** Screening workflow using our in-house database of metabolites and the R based application.

**Table S1:** Clinical data obtained from a patient’s medical records during their hospitalization at the Yale New Haven Hospital. The following clinical data were measured multiple times within the first 24 h of patient admission. For each parameter, and for each patient, a 24 h minimum value (24_hours_min) and a 24h maximum value (24_hours_max) were obtained and an average of all the measurements withing the 24h window (24_hours_mean) was calculated.

| ***Clinical parameter*** | ***Organ/system*** | ***Clinical parameter*** | ***Organ/system*** |
| --- | --- | --- | --- |
| WBC | Immune system | PAO2 | Lung (oxygen pressure in arterial blood) |
| HGB | Lung (Hemoglobin, O_2_ carrier) | PACO2 | Lung (carbon dioxide (CO2) levels in the blood) |
| PLT | Blood clotting (blood) | PH; arterial | Metabolic acidosis |
| Sodium | Kidney | APTT | Liver response (Blood clotting) |
| Chloride | Kidney | INR | Liver response (Blood clotting) |
| Calcium | Kidney | PT | Liver response (Blood clotting) |
| Potassium | Kidney failure | Fibrinogen | Liver response (Blood clotting) |
| Creatinine | Kidney failure | ESR | Blood clotting (blood) |
| Glucose | Metabolic | LDH | Tissue damage |
| Bicarbonate | Lung | RF | Immune system |
| BUN | Kidney failure | Albumin | Liver |
| Bilirubin | Liver damage (cholestatic) | Globulin | Immune system |
| ALT | Liver damage | LVEF | Heart |
| AST | Liver | TROP_T | Heart attack |
| Total protein | Chronic kidney disease | ESI | Emergency Severity Index (ESI): A Triage Tool for Emergency Departments |
| Alk Phos | Liver damage (cholangiopathy) | HR | Heart |
| Ferritin | Liver response (acute response) | RR | Respiratory rate (RR) |
| Trop I | Heart attack | Systolic | Heart |
| Dimer | Liver response (blood clotting) | Diastolic | Heart |
| Lactate | Metabolic acidosis [impaired tissue oxygenation (decreased O_2_ or mitochondrial dysfunction)] | Monocytes_abs | Immune system |
| GCS | Level of consciousness | EOS | Immune system |
| BMI | BMI | BASOS | Immune system |
| RBC | Blood | Immature granulocytes | Immune system |
| Hematocrit | Blood | Immature granulocytes_abs | Immune system |
| MCV | Blood | Metamyelocytes | Immune system |
| ***Clinical parameter*** | ***Organ/system*** | ***Clinical parameter*** | ***Organ/system*** |
| MCH | Blood | BANDS | Immune system |
| MCHC | Blood | A1C | Metabolic (diabetes) |
| RDW | Blood | Cholesterol | Viral replication (COVID-19 patients have lower cholesterol in blood but higher in tissues) |
| Neutrophils | Immune system | Antithrombin III | Blocks blood clotting |
| Lymphs | Immune system | TEMP | Temperature |
| CRP | Liver response (inflammation marker) | SPO2 | Lung |
| Procal | Bacterial infections/sepsis |  | |

**Table S2:** Comorbidities of SARS-CoV-2 infected patients when admitted to the Yale New Haven hospital**.**

| ***Comorbidities of SARS-CoV-2 infected patients*** | |
| --- | --- |
| 1 Tuberculosis | 41 Cancer; other and unspecified primary |
| 2 Septicemia (except in labor) | 42 Secondary malignancies |
| 3 Bacterial infection; unspecified site | 43 Malignant neoplasm without specification of site |
| 4 Mycoses | 44 Neoplasms of unspecified nature or uncertain behavior |
| 5 HIV infection | 45 Maintenance chemotherapy; radiotherapy |
| 6 Hepatitis | 46 Benign neoplasm of uterus |
| 7 Viral infection | 47 Other and unspecified benign neoplasm |
| 8 Other infections; including parasitic | 48 Thyroid disorders |
| 9 Sexually transmitted infections (not HIV or hepatitis) | 49 Diabetes mellitus without complication |
| 10 Immunizations and screening for infectious disease | 50 Diabetes mellitus with complications |
| 11 Cancer of head and neck | 51 Other endocrine disorders |
| 12 Cancer of esophagus | 52 Nutritional deficiencies |
| 13 Cancer of stomach | 53 Disorders of lipid metabolism |
| 14 Cancer of colon | 54 Gout and other crystal arthropathies |
| 15 Cancer of rectum and anus | 55 Fluid and electrolyte disorders |
| 16 Cancer of liver and intrahepatic bile duct | 56 Cystic fibrosis |
| 17 Cancer of pancreas | 57 Immunity disorders |
| 18 Cancer of other GI organs; peritoneum | 58 Other nutritional; endocrine; and metabolic disorders |
| 19 Cancer of bronchus; lung | 59 Deficiency and other anemia |
| 20 Cancer; other respiratory and intrathoracic | 60 Acute posthemorrhagic anemia |
| 21 Cancer of bone and connective tissue | 61 Sickle cell anemia |
| 22 Melanomas of skin | 62 Coagulation and hemorrhagic disorders |
| 23 Other non-epithelial cancer of skin | 63 Diseases of white blood cells |
| 24 Cancer of breast | 64 Other hematologic conditions |
| 25 Cancer of uterus | 76 Meningitis (except that caused by tuberculosis or sexually transmitted disease) |
| 26 Cancer of cervix | 77 Encephalitis (except that caused by tuberculosis or sexually transmitted disease) |
| 27 Cancer of ovary | 78 Other CNS infection and poliomyelitis |
| 28 Cancer of other female genital organs | 79 Parkinson`s disease |
| 29 Cancer of prostate | 80 Multiple sclerosis |
| 30 Cancer of testis | 81 Other hereditary and degenerative nervous system conditions |
| 31 Cancer of other male genital organs | 82 Paralysis |
| 32 Cancer of bladder | 83 Epilepsy; convulsions |
| 33 Cancer of kidney and renal pelvis | 84 Headache; including migraine |
| 34 Cancer of other urinary organs | 85 Coma; stupor; and brain damage |
| 35 Cancer of brain and nervous system | 86 Cataract |
| 36 Cancer of thyroid | 87 Retinal detachments; defects; vascular occlusion; and retinopathy |
| 37 Hodgkin`s disease | 89 Blindness and vision defects |
| 38 Non-Hodgkin`s lymphoma | 90 Inflammation; infection of eye (except that caused by tuberculosis or sexually transmitted disease) |
| 39 Leukemias | 91 Other eye disorders |
| 40 Multiple myeloma | 92 Otitis media and related conditions |
| 93 Conditions associated with dizziness or vertigo | 140 Gastritis and duodenitis |
| 94 Other ear and sense organ disorders | 141 Other disorders of stomach and duodenum |
| 95 Other nervous system disorders | 142 Appendicitis and other appendiceal conditions |
| 96 Heart valve disorders | 143 Abdominal hernia |
| 97 Peri-; endo-; and myocarditis; cardiomyopathy (except that caused by tuberculosis or sexually transmitted disease) | 144 Regional enteritis and ulcerative colitis |
| 98 Essential hypertension | 145 Intestinal obstruction without hernia |
| 99 Hypertension with complications and secondary hypertension | 146 Diverticulosis and diverticulitis |
| 100 Acute myocardial infarction | 147 Anal and rectal conditions |
| 101 Coronary atherosclerosis and other heart disease | 148 Peritonitis and intestinal abscess |
| 102 Nonspecific chest pain | 149 Biliary tract disease |
| 103 Pulmonary heart disease | 150 Liver disease; alcohol-related |
| 104 Other and ill-defined heart disease | 151 Other liver diseases |
| 105 Conduction disorders | 152 Pancreatic disorders (not diabetes) |
| 106 Cardiac dysrhythmias | 153 Gastrointestinal hemorrhage |
| 107 Cardiac arrest and ventricular fibrillation | 154 Noninfectious gastroenteritis |
| 108 Congestive heart failure; nonhypertensive | 155 Other gastrointestinal disorders |
| 109 Acute cerebrovascular disease | 156 Nephritis; nephrosis; renal sclerosis |
| 110 Occlusion or stenosis of precerebral arteries | 158 Chronic kidney disease |
| 111 Other and ill-defined cerebrovascular disease | 159 Urinary tract infections |
| 112 Transient cerebral ischemia | 160 Calculus of urinary tract |
| 113 Late effects of cerebrovascular disease | 161 Other diseases of kidney and ureters |
| 114 Peripheral and visceral atherosclerosis | 162 Other diseases of bladder and urethra |
| 115 Aortic; peripheral; and visceral artery aneurysms | 163 Genitourinary symptoms and ill-defined conditions |
| 116 Aortic and peripheral arterial embolism or thrombosis | 164 Hyperplasia of prostate |
| 117 Other circulatory disease | 165 Inflammatory conditions of male genital organs |
| 118 Phlebitis; thrombophlebitis and thromboembolism | 166 Other male genital disorders |
| 119 Varicose veins of lower extremity | 167 Nonmalignant breast conditions |
| 120 Hemorrhoids | 168 Inflammatory diseases of female pelvic organs |
| 121 Other diseases of veins and lymphatics | 169 Endometriosis |
| 122 Pneumonia (except that caused by tuberculosis or sexually  transmitted disease) | 170 Prolapse of female genital organs |
| 123 Influenza | 171 Menstrual disorders |
| 124 Acute and chronic tonsillitis | 172 Ovarian cyst |
| 125 Acute bronchitis | 173 Menopausal disorders |
| 126 Other upper respiratory infections | 174 Female infertility |
| 127 Chronic obstructive pulmonary disease and bronchiectasis | 175 Other female genital disorders |
| 128 Asthma | 171 Menstrual disorders |
| 129 Aspiration pneumonitis; food/vomitus | 172 Ovarian cyst |
| 130 Pleurisy; pneumothorax; pulmonary collapse | 173 Menopausal disorders |
| 131 Respiratory failure; insufficiency; arrest (adult) | 174 Female infertility |
| 132 Lung disease due to external agents | 175 Other female genital disorders |
| 133 Other lower respiratory disease | 176 Contraceptive and procreative management |
| 134 Other upper respiratory disease | 177 Spontaneous abortion |
| 135 Intestinal infection | 178 Induced abortion |
| 136 Disorders of teeth and jaw | 179 Postabortion complications |
| 137 Diseases of mouth; excluding dental | 180 Ectopic pregnancy |
| 138 Esophageal disorders | 181 Other complications of pregnancy |
| 139 Gastroduodenal ulcer (except hemorrhage) | 182 Hemorrhage during pregnancy; abruptio placenta; placenta previa |
| 183 Hypertension complicating pregnancy; childbirth and the  puerperium | 226 Fracture of neck of femur (hip) |
| 184 Early or threatened labor | 227 Spinal cord injury |
| 185 Prolonged pregnancy | 229 Fracture of upper limb |
| 186 Diabetes or abnormal glucose tolerance complicating  pregnancy; childbirth; or the puerperium | 230 Fracture of lower limb |
| 187 Malposition; malpresentation | 231 Other fractures |
| 188 Fetopelvic disproportion; obstruction | 232 Sprains and strains |
| 189 Previous C-section | 233 Intracranial injury |
| 190 Fetal distress and abnormal forces of labor | 234 Crushing injury or internal injury |
| 191 Polyhydramnios and other problems of amniotic cavity | 235 Open wounds of head; neck; and trunk |
| 192 Umbilical cord complication | 236 Open wounds of extremities |
| 193 OB-related trauma to perineum and vulva | 237 Complication of device; implant or graft |
| 194 Forceps delivery | 238 Complications of surgical procedures or medical care |
| 195 Other complications of birth; puerperium affecting  management of mother | 239 Superficial injury; contusion |
| 196 Other pregnancy and delivery including normal | 240 Burns |
| 197 Skin and subcutaneous tissue infections | 241 Poisoning by psychotropic agents |
| 198 Other inflammatory condition of skin | 242 Poisoning by other medications and drugs |
| 199 Chronic ulcer of skin | 243 Poisoning by nonmedicinal substances |
| 202 Rheumatoid arthritis and related disease | 244 Other injuries and conditions due to external causes |
| 203 Osteoarthritis | 245 Syncope |
| 204 Other non-traumatic joint disorders | 246 Fever of unknown origin |
| 205 Spondylosis; intervertebral disc disorders; other back  problems | 247 Lymphadenitis |
| 206 Osteoporosis | 248 Gangrene |
| 207 Pathological fracture | 249 Shock |
| 208 Acquired foot deformities | 250 Nausea and vomiting |
| 209 Other acquired deformities | 251 Abdominal pain |
| 210 Systemic lupus erythematosus and connective tissue  disorders | 252 Malaise and fatigue |
| 211 Other connective tissue disease | 253 Allergic reactions |
| 212 Other bone disease and musculoskeletal deformities | 254 Rehabilitation care; fitting of prostheses; and adjustment of devices |
| 213 Cardiac and circulatory congenital anomalies | 255 Administrative/social admission |
| 214 Digestive congenital anomalies | 256 Medical examination/evaluation |
| 215 Genitourinary congenital anomalies | 257 Other aftercare |
| 216 Nervous system congenital anomalies | 258 Other screening for suspected conditions (not mental disorders or infectious disease) |
| 217 Other congenital anomalies | 259 Residual codes; unclassified |
| 218 Live born | 260 E Codes: All (external causes of injury and poisoning) |
| 219 Short gestation; low birth weight; and fetal growth  retardation | 651 Anxiety disorders |
| 220 Intrauterine hypoxia and birth asphyxia | 652 Attention-deficit, conduct, and disruptive behavior disorders |
| 221 Respiratory distress syndrome | 653 Delirium, dementia, and amnestic and other cognitive disorders |
| 222 Hemolytic jaundice and perinatal jaundice | 654 Developmental disorders |
| 223 Birth trauma | 655 Disorders usually diagnosed in infancy, childhood, or adolescence |
| 224 Other perinatal conditions | 656 Impulse control disorders, NEC |
| 225 Joint disorders and dislocations; trauma-related | 657 Mood disorders |
| 226 Fracture of neck of femur (hip) | 658 Personality disorders |
| 659 Schizophrenia and other psychotic disorders |  |
| 660 Alcohol-related disorders |  |
| 661 Substance-related disorders |  |
| 662 Suicide and intentional self-inflicted injury |  |
| 2601 E Codes: Cut/pierce b |  |
| 2602 E Codes: Drowning/submersion |  |
| 2604 E Codes: Fire/burn |  |
| 2602 E Codes: Drowning/submersion |  |
| 2604 E Codes: Fire/burn |  |
| 2605 E Codes: Firearm |  |
| 2606 E Codes: Machinery |  |
| 2607 E Codes: Motor vehicle traffic (MVT) |  |
| 2608 E Codes: Pedal cyclist; not MVT |  |
| 2609 E Codes: Pedestrian; not MVT |  |
| 2610 E Codes: Transport; not MVT |  |
| 2611 E Codes: Natural/environment |  |
| 2612 E Codes: Overexertion |  |
| 2614 E Codes: Struck by; against |  |
| 2616 E Codes: Adverse effects of medical care |  |
| 2617 E Codes: Adverse effects of medical drugs |  |
| 2618 E Codes: Other specified and classifiable |  |
| 2619 E Codes: Other specified; NEC |  |
| 2620 E Codes: Unspecified |  |
| 2621 E Codes: Place of occurrence |  |
